# Bacterial quorum-sensing systems in cell–cell communication

**DOI:** 10.1093/femsre/fuag020

**Published:** 2026-05-11

**Authors:** Kai-Zhong Xu, Annadurai Vinothkanna, DanDan Wang, Hongyan Jiang, Zhenbo Xu, Ai-Qun Jia

**Affiliations:** College of Food Engineering, Zhangzhou Institute of Technology, Zhangzhou363000, China; Hainan General Hospital, Hainan Affiliated Hospital of Hainan Medical University, Haikou570311, China; Hainan General Hospital, Hainan Affiliated Hospital of Hainan Medical University, Haikou570311, China; Hainan General Hospital, Hainan Affiliated Hospital of Hainan Medical University, Haikou570311, China; Hainan General Hospital, Hainan Affiliated Hospital of Hainan Medical University, Haikou570311, China; Department of Laboratory Medicine, the Second Affiliated Hospital of Shantou University Medical College, Shantou515041, China; Hainan General Hospital, Hainan Affiliated Hospital of Hainan Medical University, Haikou570311, China

**Keywords:** bacteria, quorum-sensing systems, cell–cell communication, antimicrobial drug resistance, signaling molecules

## Abstract

Antimicrobial resistance (AMR) and the emergence of multidrug-resistant pathogens necessitate holistic care. Novel antimicrobial drug discovery involves an in-depth assessment of quorum sensing (QS) signaling and cell–cell communication. Bacteria regulate their metabolism to cope with complex host–environmental changes through QS signaling. Previous studies suggest that the social behaviors of bacteria include biofilm formation, virulence, and drug resistance mediated by QS. Over several decades, autoinducer receptors, signaling pathways, and the regulatory networks that control gene expression have corroborated QS signaling molecules. Multiple QS systems and chemical structural diversity signaling molecules have been sporadically reported, but there is no comprehensive review of these findings. This review systematically and comprehensively summarizes the paper, addressing bacterial QS-based cell–cell communication and the potential mechanisms of QS systems in bacterial drug resistance. Furthermore, a status quo update has been established for novel QS systems in the realms of pathogenicity, polymicrobial interactions, and/or AMR patterns. Nevertheless, the applications of QS systems would invoke newer incorporations for futuristic research values. Thus, the complexity and evolutionary insights of QS systems, quorum-sensing signaling molecules, and regulatory mechanisms need a “cloud” based repurposing for the design of novel agents in AMR and communication.

## Teaser

This review systematically and comprehensively summarizes the bacterial quorum sensing (QS)-based cell–cell communication and the potential mechanisms of QS systems in bacterial drug resistance.

## Introduction

Prokaryotic microorganisms, once thought of as solitary entities, have traditionally been perceived as lacking social behavior. However, evidence from community interactions, intercellular communication, and signaling cascade mechanisms confirms the existence of environmental signal transduction and social community information exchange (Kareb and Aïder 2020). Bacteria communicate through specific signaling molecules to adapt to complex ecological stress modalities. Bacterial populations exhibit a range of social behaviors, including cooperation, division of labor, antagonism, and inhibition (Wong et al. 2020). Bacterial communication includes quorum sensing (QS), intercellular contact, biofilm formation, antimicrobial activity, and other mechanisms. QS signaling mechanisms have been stressed for multicentered approaches. Research to this day has elucidated the concrete phenomenon of QS and biofilm formation in antimicrobial resistance (AMR) mechanisms.

QS refers to bacterial behaviors that involve changes in cell density, which regulate gene expression (Paul et al. 2018). The QS systems of bacteria are usually composed of signaling molecules, signaling synthases, and receptor proteins. Signaling molecules, synthesized by signaling molecule synthases, are secreted into the environment during bacterial growth. Receptor proteins bind with signaling molecules, which further regulate gene expression. This regulatory cascade is triggered once the concentration of signaling molecules reaches a specific threshold, typically in the nanomolar to micromolar range for AHLs (e.g. ∼10 nM 3OC6-HSL in *Vibrio fischeri*) (Fuqua et al. 1994). Ample research has proved that virulence factors, biofilm formation, and AMR are related to the QS systems (Liu et al. 2012, Huang et al. 2020). Several reviews have focused on various QS mechanisms involved in bacterial cell–cell communication and cross-talk. Here, this review provides a comprehensive update on QS signaling mechanisms involved in bacterial resistance. The hope is that the information will aid researchers in developing multidrug resistance platforms for the multicentered eradication of bacterial biofilms.

Since the first report of QS in 1970, large numbers of QS systems and signaling molecules have been discovered and intricate mechanisms of action have been deciphered (Yi et al. 2021). However, few comprehensive reviews have summarized different QS systems and intrinsic environmental adaptations. Furthermore, the nomenclature of QS systems and related functional roles pose this paper as novel and innovative. Here, the newly reported QS signaling molecules and their potential biosynthetic pathways, as well as the relationship between QS mechanisms and drug resistance, are discussed. Bacterial QS systems and potential targets for nonclassical antimicrobiological drug development are also illustrated. Although QS signaling molecules in microbial resistance and biofilm formation are reported, the outlook for future research remains inconclusive. Quorum quenching (QQ) has been advocated for degrading QS molecules, inhibiting receptor binding, and targeting antibodies to block QS (Tripathi et al. 2022). Hence, the cell–cell communication mechanisms need to be compiled for assessing gene interactions and metabolic signaling molecules. Recently, paecilomycone was shown to inhibit the 3,4-dihydroxy-2-heptylquinoline (the *Pseudomonas* quinolone signal, PQS) pathway of QS in Gram-negative bacteria, addressing QS targets for effective biofilm inhibition (Beenker et al. 2023). Several QS signalling mechanisms have been vigorously investigated for effective abatement of disease resistance mediated by QS. Table 1 lists the QS signalling molecules reported so far. This review aims to bridge the current gap by providing a comprehensive and analytical synthesis of bacterial QS. The discussion begins with a deconstruction of the chemical diversity and unifying biosynthetic principles of quorum-sensing signal molecules (QSSMs), followed by an analysis of the architectural and evolutionary themes in QS receptor and signal transduction networks. Building on this foundation, the review elucidates how these integrated systems orchestrate key social behaviors, including biofilm formation, virulence, and AMR. Finally, the mechanistic understanding is translated to the clinical arena by examining the role of QS in specific high-priority pathogens and within the complex ecosystem of polymicrobial infections. By adopting this integrative framework, the discussion moves beyond a descriptive catalog of systems toward a critical appraisal of QS as a central and targetable regulator of bacterial community pathogenicity.

**Table 1:** QS signaling molecules of various pathogens.

Pathogens	Signaling molecules	Structures	Mechanism of action	Known QSIs/QQ agents	References
Gram-negative					
*V. fischeri* and *P. aeruginosa*	Acyl-homoserine lactones	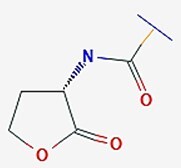	Population density-based cell-to-cell communication using diffusible signals in a heterogeneous and spatial community.	Fusaric acid, phenazine carboxylic acid, Citrinin, and AiiA.	Tung et al. (2017), Patel et al. (2020), Qin et al. (2020), Ji et al. (2023), Rosic et al. (2025)
*E. coli* and *Salmonella* sp.	3,6-dimethylpyrazin-2(1*H*)-one	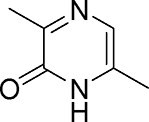	Decreased toxicity and global regulation of *Vp*_AHPND_ infection using QseC with reduced virulence	Fructose furoic, isolimonic acid, and esculetin	
*E. coli* and *Salmonella* sp.	Epinephrine	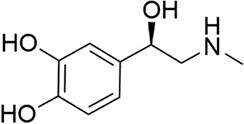	Decreased toxicity and global regulation of *Vp*_AHPND_ infection using QseC with reduced virulence		Jeon and Itoh (2007), Vikram et al. (2012), Vinothkannan et al. (2018), Yang et al. (2020), Mi et al. (2024)
*E. coli* and *Salmonella* sp.	Norepinephrine	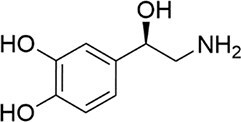	Decreased toxicity and global regulation of *Vp*_AHPND_ infection using QseC with reduced virulence		
*P. aeruginosa*	2-heptyl-4-quinolone	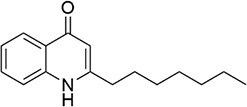	Complex QS regulation by differential regulation of *phz1* and *phz2* phenazine operons	Quinazolinone disulfide analogues, Clofoctol	D’Angelo et al. (2018), Higgins et al. (2018), Sabir et al. (2023)
*P. aeruginosa*	2-heptyl-3-hydroxy-4-quinolone	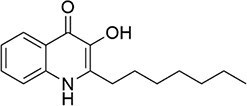	Complex QS regulation by differential regulation of *phz1* and *phz2* phenazine operons		
*V. cholera* and *Photobacteria* sp.	(*S*)-3-hydroxytridecan-4-one	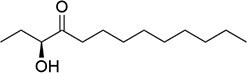	QSSM signal–receptor specificity for enhanced virulence	Isonaamidine A, isonaamine D	Ke et al. (2014), Mai et al. (2015)
*V. cholerae*	3,5-dimethylpyrazin-2-ol	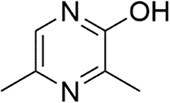	Biofilm formation and toxin production by increased small RNA regulation	To be discovered	Papenfort et al. (2017)
*V. harveyi*	*S*-THMF-borate	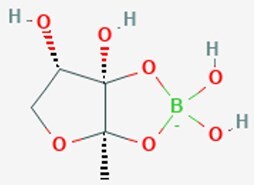	Presence of boron for naturally occurring borate to AI-2 precursor, resulting in active AI-2	Moracin M, cannabigerolic acid, Piperine,	Xin et al. (2002), Arcan et al. (2025, Helcman et al. (2025)
*V. harveyi*	*R*-THMF	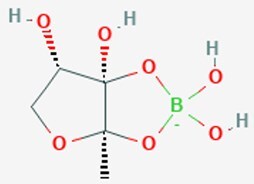	Presence of boron for naturally occurring borate to AI-2 precursor, resulting in active AI-2		Xin et al. (2002)
*X. campestris pv. campestris*	Diffusible signaling factor	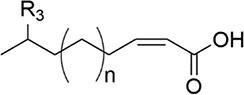	Biofilm formation architecture, antibiotic resistance, and increased virulence	Zingerone derivatives	Liang et al. (2025)
*S. baltica*	2,5-diketopiperazines	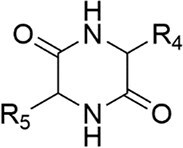	Positive transcription regulation leading to food preservation avoiding spoilage	To be discovered	Wang et al. (2019)
*S. sonnei*	4-hydroxybenzoic acid	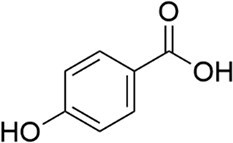	Competitive QSSMs for interkingdom communication	To be discovered	Wang et al. (2023a)
*P. aeruginosa*	2’-amino acetophenone	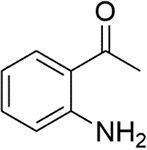	Novel antioxidant signal cascade for muscle dysfunction	To be discovered	Bandyopadhaya et al. (2019)
*P. sympiotica*	dDialkylresorcinols	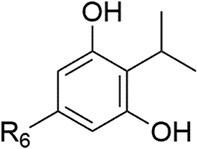	Novel QSSMs for escalated virulence	To be discovered	Brameyer et al. (2015)
*P. luminescens*	Photopyrone	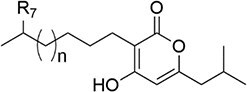	Biosynthesis of antibiotics like myxopyronin and corallopyronin	To be discovered	Kresovic et al. (2015)
*R. solanacearum*	3-hydroxy-palmitic acid methyl ester	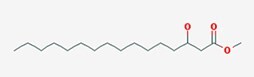	QQ against wilt prevention	ELP86, ELP96, ELP104, and EstDL33	Achari and Ramesh (2015), Lee et al. (2018)
Gram-positive					
*Streptomyces* spp.	*γ*-butyrolactones	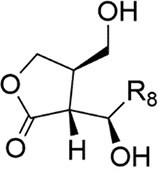	QSSMs in γ-butyrolactone (GBL) and acyl-homoserine lactone cross-talk	To be discovered	Liu et al. (2021)
*S. aureus*	Autoinducer peptide-I	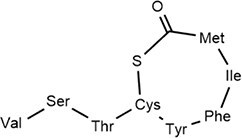	Peptide signaling in QS system	Cyclodepsipeptides-WS9326A, WS9326B and cochinmicin II/III	Matthew Thoendel et al. (2011), Desouky et al. (2015)
*S. aureus*	Autoinducer peptide-Ⅱ	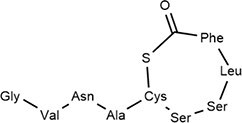	Peptide signaling in QS system		Matthew Thoendel et al. (2011)
*B. subtilis*	Autoinducer peptide -Ⅲ	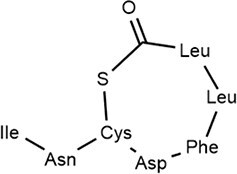	Competence pheromone-induced genetic polymorphism and specificity of QS mechanism	*N*-(pyridin-2-yl)-benzamides derivatives	Tortosa et al. (2001)
*S. aureus*	Autoinducer peptide -Ⅳ	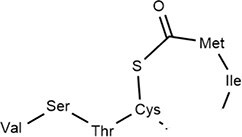	Peptide signaling in QS system	Immersing a nitrocellulose	Matthew Thoendel et al. (2011) (Inagaki et al. (2024)
*L. monocytogenes*	Indole	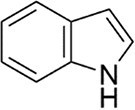	Indole signaling and gut microbiota homeostasis	Carnosol	Rattanaphan et al. (2020), Cui et al. (2025)

## Chemical diversity and biosynthetic logic of QS signaling molecules

Bacterial communication operates through a diverse chemical lexicon. QSSMs encompass multiple structural classes—including fatty acid derivatives, peptides, and modified amino acids—each biosynthesized via dedicated enzymatic pathways. Deciphering this chemical vocabulary and its underlying biosynthetic logic constitutes the essential foundation for understanding QS-mediated social behaviors. A consolidated overview of the major QSSM classes, their structures, and representative producer organisms is provided in Table 1.

### *N*-acyl homoserine lactones: the prototypical Gram-negative signals

**Figure 1:**
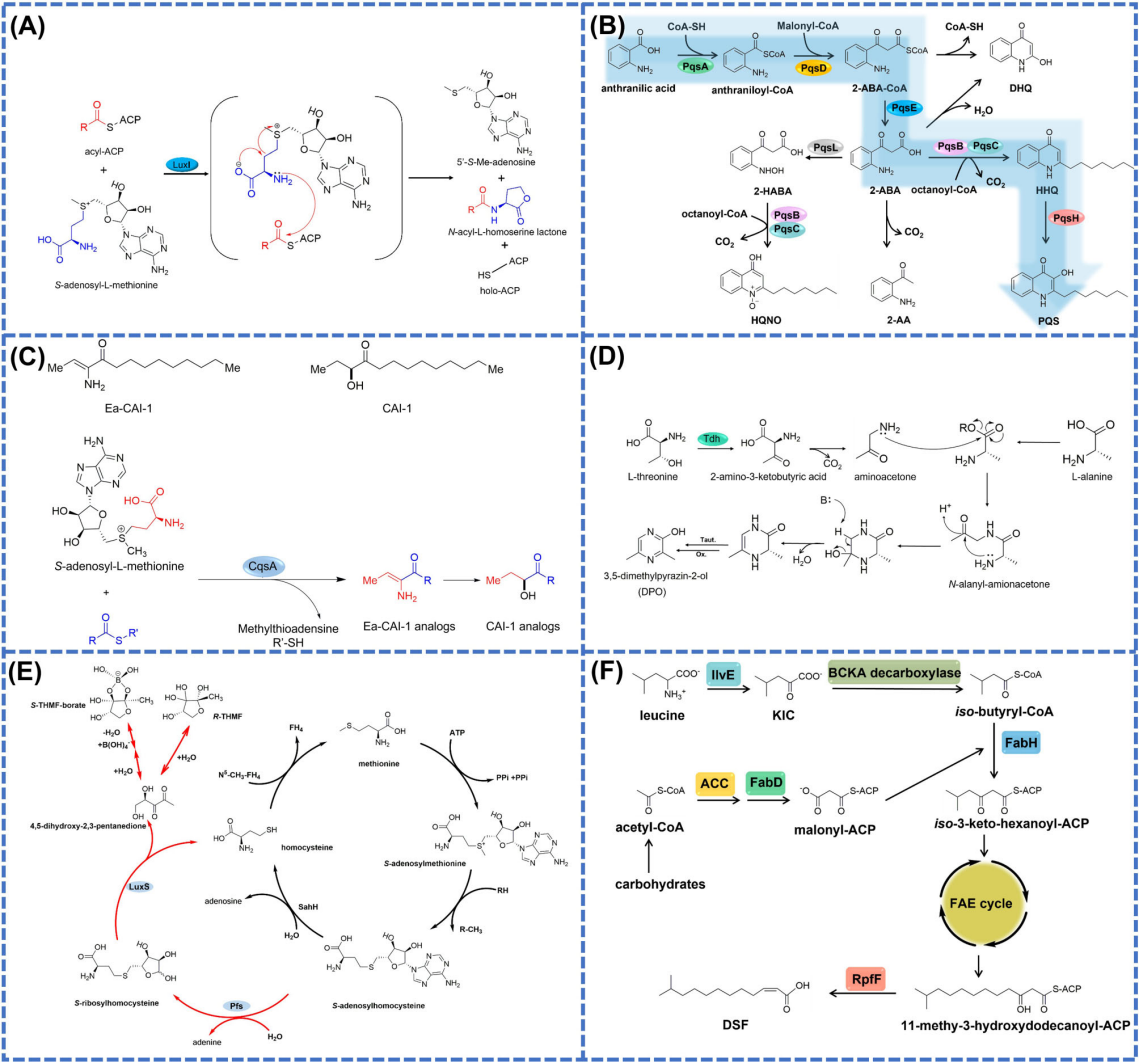
Biosynthetic pathways of six representative QS signaling molecules. (A) AHL biosynthesis via LuxI/LuxM/HdtS pathways. (B) Alkyl-quinolone (PQS/HHQ) synthesis from anthranilic acid. (C) CAI-1 synthesis by CqsA from aminotransferase activity. (D) DPO synthesis from l-threonine via Tdh and condensation steps. (E) AI-2 synthesis from SAM via LuxS and Pfs enzymes. (F) DSF synthesis via fatty acid synthesis and RpfF activity.

*N*-acyl homoserine lactones (AHLs) are some of the most extensively studied signaling molecules. AHLs are involved in QS as signaling molecules of most Gram-negative bacteria. The first AHLs reported (in 1981) were produced by *V. fischeri*, a marine bacterium commonly found in seafood, which is of particular concern in food safety due to its potential involvement in spoilage and contamination (Patel et al. 2020). Subsequently, AHLs bearing different amide chains were identified in many Gram-negative bacteria such as *Pseudomonas aeruginosa*, *Shewanella baltica*, and *Aeromonas hydrophila* (Jin et al. 2020). Structurally, the backbone of AHLs consists of a homoserine lactone ring and an amide bond (Table 1). Variations in the acyl chain length (C4–C18) and substitutions (e.g. hydroxyl, oxo, hydrogen, and carbonyl) at the third carbon or other positions in the amide chain influence bacterial signal specificity and perception. The biosynthesis of AHLs depends on AHL-related synthetases in bacteria. Currently, the reported AHL synthetases in Gram-negative bacteria primarily include LuxI, LuxM, and HdtS (Table 2). LuxI-type synthases (e.g. LuxI, LasI, and RhlI) utilize *S*-adenosylmethionine (SAM) and an acyl-acyl carrier protein as substrates, coupling acylation and lactonization to produce AHLs like 3-oxo-C6-HSL in *V. fischeri* (Fig. 1A) and 3-oxo-C12-HSL/C4-HSL in *P. aeruginosa* (Kumar et al. 2022). LuxM-type synthases (e.g. LuxM and VanM) were first identified in *V. harveyi* and are responsible for synthesizing signals like 3-OH-C4-HSL (Jiang. et al. 2014). They represent an evolutionarily distinct family from LuxI (Milton et al. 2001, Fang et al. 2018). HdtS-type synthases exhibit dual activity, functioning in both lysophosphatidic acid acylation and AHL synthesis (e.g. C6-HSL and 3-OH-C14-HSL) in bacteria like *P. fluorescens* and *Acidithiobacillus ferrooxidans* (Laue et al. 2000, Jiang. et al. 2014, Condori et al. 2016). The coexistence of multiple AHL synthase families in nature highlights the evolutionary convergence on AHLs as effective signaling molecules.

**Table 2:** QS systems based on acyl-homoserine lactones (AHLs) and autoinducer peptides (AIPs) of typical Gram-negative and Gram-positive pathogens.

Typical species	QS systems	Signaling molecules	Functional roles	References
Gram-negative				
*V. fischeri*	LuxI/LuxR	3-oxo-C6-HSL	Biofilm formation and bioluminescence	Patel et al. (2020)
*P. aeruginosa*	LasI/LasR	3-oxo-C12-HSL	Elastase,	Schutz and Empting (2018)
	RhlI/RhlR	C4-HSL	rhamnolipids, and pyocyanin	
*C. violaceum*	CviI/CviR	C6-HSL	Violacein, hemolysin, and chitinase	Cheng et al. (2020)
*B. cenocepacia*	CepI/CepIR	C8-HSL	Proteases,siderophores and biofilm formation	Ryan et al. (2013)
*A. hydrophila*	AhyI/AhyR	C4-HSL, C6-HSL	Biofilm formation, hemolysin production and motility	Jin et al. (2020)
*E. carotovora*	ExpI/ExpRCarI/CarR	3-oxo-C6-HSL,C7-HSL	Produce of plant cell wall-degrading enzymes and carbapenem production	Vieira et al. (2020)
*R. solanacearum*	SolI/SolR	C6-HSL, C8-HSL	Virulence	Chen et al. (2009)
*A. salmonicida*	AsaI/AsaR	C4-HSL	Spoilage, motility and biofilm formation	Liu et al. (2018)
*S. marcescens*	SwrI/SwrRSpnI/SpnR	C4-HSL, C6-HSL	Sliding, synthesis of biosurfactant, prodigiosin, and nuclease	Zhou et al. (2019)
*V. parahaemolyticus*	LuxM/LuxN	*N*-(3-hydroxybutanoyl)-HSL	*N*-(3-hydroxybutanoyl)-HSL	Henke and Bassler (2004)
*V. anguillarum*	VanI/VanR VanM/VanN	C6-HSL and OH-C6-HSL	eExtracellular proteases and biofilm formation	Buchholtz et al. (2006)
*P. fluorescens*	HdtS/?	C6-HSL, C10-HSL, and N–(3 -OH-7-*cis*-tetradecenoyl)-HSL	Unknown	Laue et al. (2000)
Gram-positive				
Typical species	QS systems	Signaling molecules	Functional roles	References
*S. aureus*	AgrD/AgrCA	AgrD	*α*-hemolysin, *δ*-toxin and other virulence factors	Xie et al. (2019)
*S. pneumoniae *	TprA/PhrA	TprA	Lantibiotic and galactose metabolism	Hoover et al. (2015)
	ComC/ComDE	ComC	Biofilm formation and virulence	Shanker and Federle (2017)
*S. thermophilus*	BlpC/BlpHR	BlpC	Bacteriocin production	Renye and Somkuti (2013)
*B. subtilis*	ComX/ComPA	ComX	Influence sporulation, competence, and biofilm development	Spacapan et al. (2020)
*L. lactis*	ComX/ComPA	ComX	The acid tolerance and nisin yield	Yuan et al. (2021)
*P. polymyxa*	AloP/AloR	AloP	Remains to be elucidated	Hoover et al. (2015)
*B. thuringiensis*	PapR/PlcR	PapR	Cytotoxins, degradative enzymes and surface proteins	Huillet et al. (2020)
*E. faecalis*	FsrD/FsrC	FsrD	Biofilm formation and virulence	McBrayer et al. (2018)
*B. subtilis*	PhrA/RapAPhrB/RapBPhrP/RapPPhrH/RapH	PhrAPhrBPhrPPhrH	Biofilm formation and plant root colonization	Nordgaard et al. (2021)
*B. cereus*	NprX/NprR	NprX	Necrotrophic and spore formation	Zouhir et al. (2013)
*B. thuringiensis*	PapR/PlcR	PapR	Virulence	Slamti et al. (2016)
*E. faecalis*	cCF10/PrgXiCF10/PrgX	ccF10iCF10	Conjugation inhibited or induced	Chen et al. (2017)
*S. pyogenes*	SHP2/Rgg2SHP3/Rgg3	SHP2SHP3	Transcriptional repressor and controlling surface attributes	Aggarwal et al. (2014)

### Peptide-based signals: the Gram-positive paradigm and beyond

Gram-positive bacteria predominantly utilize autoinducing peptides (AIPs) as QSSMs. These are typically synthesized as precursor peptides (pre-AIPs) on ribosomes and then enzymatically processed, often involving cyclization (e.g. thiolactone rings in *S. aureus* AIPs) to generate the mature, active signal (Hoover et al. 2015, Reuter et al. 2016). The *agr* system of *Staphylococcus aureus* serves as the archetype: AgrD encodes the pre-AIP, which is processed and exported by AgrB, and then sensed by the membrane-bound histidine kinase AgrC (Li et al. 2022). Another major class of peptide signals is recognized by the intracellular RRNPP family receptors (named for Rap, Rgg, NprR, PlcR, and PrgX) (Neiditch et al. 2017). These peptides, such as NprX in *B. cereus*, are imported into the cell via oligopeptide permease (Opp) systems before binding their cytosolic receptors (Zouhir et al. 2013).Many studies have characterized the diverse structure of AIP signaling molecules (Gless et al. 2017). AIPs are generally composed of 5–17 amino acid residues with side chains of AIPs containing isopentane and thiolactone rings. Several typical AIPs (AIP-I, AIP-Ⅱ, AIP-Ⅲ, and AIP-Ⅳ) have been reported (Table 1). This fundamental difference—membrane-bound vs. intracellular perception—reflects a key architectural divergence in QS circuitry between different Gram-positive bacteria.

AIP-based QS systems have been reported in almost all Gram-positive bacteria, based on two-component regulatory systems (Table 2) (Yuan et al. 2021). The synthesis of the AIP precursor peptide (pre-AIP) occurs during the growth of Gram-positive bacteria (Li et al. 2022). The Pre-AIP needs to be processed and modified to become an active AIP. In *S. aureus*, it is encoded by the *agrD* gene. Pre-AIP is processed into mature AIP by AgrB (membrane protein) and secreted outside cells with the help of SpsB (signal peptidase) and AgrB.

### Universal and interspecies signals: AI-2 and its perception diversity

AI-2 is postulated to serve as a universal signal for interspecies communication, a function that underscores its distinctive status (Mir and a et al. 2019). Its biosynthesis follows a conserved pathway derived from the activated methyl cycle: *S*-adenosylhomocysteine is first converted to *S*-ribosylhomocysteine (SRH) by the enzyme Pfs, after which LuxS cleaves SRH to yield 4,5-dihydroxy-2,3-pentanedione (DPD). DPD subsequently undergoes spontaneous rearrangement into multiple biologically active AI-2 isomers (Fig. 1E)(Mandabi et al. 2015, Mina and Chbib 2019). Notably, the perception of AI-2 is not universal but is mediated through at least three evolutionarily independent receptor systems, reflecting functional diversification of this conserved molecule. In *Vibrio* species, AI-2 is detected via the LuxP/LuxQ system, where LuxP specifically binds a boron-complexed derivative of DPD (*S*-THMF-borate) and interacts with the membrane-associated histidine kinase LuxQ (Xin et al. 2002). In Enterobacteriaceae such as *Escherichia coli* and *Salmonella*, the LsrB/Lsr system predominates: LsrB recognizes a non-boronated form (*R*-THMF), leading to active import of AI-2, phosphorylation by LsrK, and subsequent binding to the transcriptional repressor LsrR, thereby derepressing the lsr operon (Zhang et al. 2017, Fan et al. 2022). More recently, a third widespread mechanism has been identified involving dCACHE (double Cache)-domain-containing receptors. These membrane-integrated sensors, exemplified by KinD in *B. subtilis* and PctA in *P. aeruginosa*, bind AI-2 directly via their extracellular dCACHE domains, unveiling a phylogenetically dispersed yet previously unrecognized paradigm for AI-2 sensing (Dago et al. 2012, Zhang et al. 2020, Liu et al. 2022).

### Amino acid-derived signals: AI-3 structure and biosynthesis

**Figure 2:**
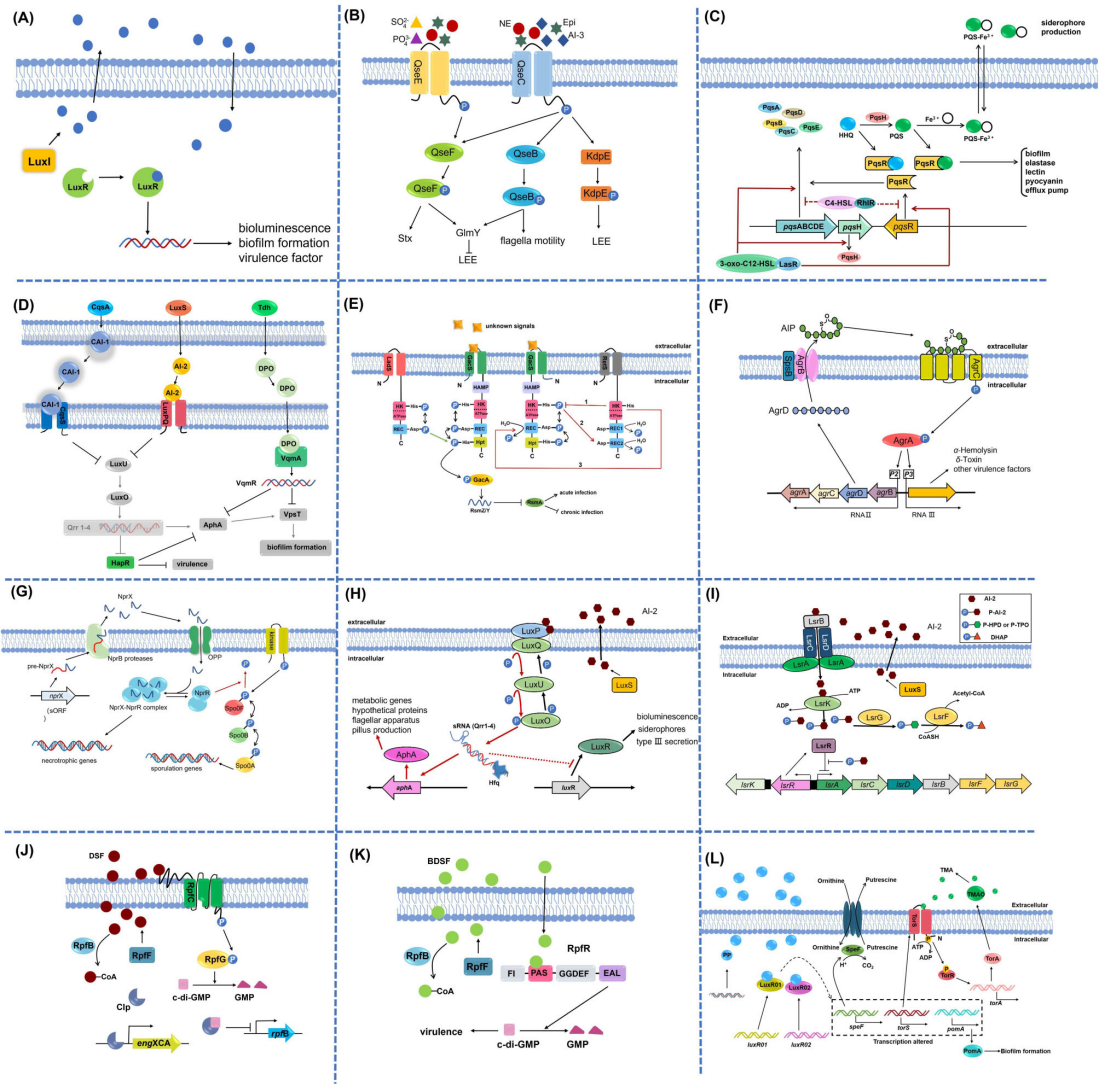
Regulatory mechanisms of six representative QS systems mediated by their receptors and signaling molecules. (A) LuxI/LuxR system regulating lux gene expression. (B) AI-3/QseCB system modulating virulence and motility in EHEC. (C) Hierarchical regulation of Las, Rhl, and Pqs systems in *P. aeruginosa*. (D) CAI-1/CqsS and DPO/VqmA systems in *V. cholerae*. (E) GacS/GacA system integrating environmental signals via sncRNAs. (F) AIP/Agr system regulating virulence in *S. aureus*. (G) RRNPP system-mediating peptide signaling in *Bacillus*. (H) AI-2/LuxPQ system in *Vibrio* spp. (I) AI-2/Lsr system in *E. coli* and *Salmonella*. (J) DSF/RpfC-RpfG system in *Xanthomonas*. (K) DSF/RpfR system in *Burkholderia*. (L) DKP/LuxR system in *S. baltica*.

Another significant class of amino acid-derived signaling molecules is autoinducer-3 (AI-3). AI-3 was found in 2003 in enterohemorrhagic *E. coli* (Fig. 2B) and later in many Gram-negative bacteria (*Salmonella* and *A. hydrophila* etc.) (Cao et al. 2019). Its chemical structure has now been elucidated, belonging to a family of pyrazinone derivatives with the primary active form identified as 3,6-dimethylpyrazin-2(1*H*)-one (Kim et al. 2020). This discovery emerged from the application of cellular stress-induced metabolite stimulation techniques and high-resolution ‌liquid chromatography-mass spectrometry (LC-MS) analysis, which led to the isolation and structural elucidation of a family of related pyrazinone analogs from *E. coli* cultures. The biosynthesis of AI-3 is directly linked to central amino acid metabolism, originating from l-threonine. The key enzyme initiating the pathway is threonine dehydrogenase (Tdh), which catalyzes the oxidation of l-threonine. A fascinating and more complex biosynthetic picture has emerged. Beyond the simple Tdh-mediated route, studies indicate that AI-3 analog formation involves a two-step spontaneous chemical process. First, Tdh-derived intermediates, such as aminoacetone (from l-threonine) or 1-amino-3-methylbutan-2-one (from 3R-hydroxy-l-leucine), spontaneously condense with aminoacyl-adenylate intermediates. These aminoacyl-AMPs are generated by “abortive” charging reactions of aminoacyl-tRNA synthetases in the absence of their cognate tRNAs. The resulting linear dipeptide ketones then undergo spontaneous cyclization, dehydration, and oxidation to yield the final pyrazinone structures, including the active AI-3 form (Kim et al. 2020). This unique mechanism underscores how bacteria co-opt core metabolic and translational machinery to generate specialized communication signals, representing an efficient evolutionary strategy. The use of “abortive” tRNA synthetase products also directly links AI-3 production to cellular metabolic and translational stress. The discovery of AI-3’s biosynthetic origin from a primary metabolic pathway reveals a profound metabolic link between bacterial signaling and fundamental physiology. This connection is particularly significant given that AI-3 shares functional similarities with host catecholamines, epinephrine (Epi) and norepinephrine (NE), and is recognized by the same bacterial membrane sensor kinase (QseC) in EHEC, facilitating interkingdom signaling (Cao et al. 2019). Recent transcriptomic studies in *S. Typhimurium* indicate that AI-3 triggers broad regulatory responses, often repressing genes involved in iron acquisition, gut colonization, and biofilm formation while upregulating certain adhesion factors (Lallement et al. 2023).

### Specialized and emerging signaling molecules

Beyond the major classes discussed above, bacteria employ a diverse array of specialized QSSMs, often uniquely adapted to specific ecological niches. These molecules exhibit distinct chemical structures, and their biosynthetic pathways are frequently intertwined with core or specialized metabolic networks. The structural specificity of these QSSMs dictates their recognition and functional outcomes in bacterial communication.

Alkyl-quinolones (AQs): AQ-type signals are particularly significant in bacteria like *P. aeruginosa*. The primary signals are 2-heptyl-3-hydroxy-4-quinolone (PQS) and its immediate precursor, 2-heptyl-3-hydroxy-4-quinoline (HHQ) (Sams et al. 2016). Their biosynthesis originates from anthranilic acid, a metabolite of tryptophan catabolism. In *P. aeruginosa*, the *pqsABCD* gene cluster encodes enzymes that convert anthranilic acid to HHQ, which is secreted (Fig. 1B). HHQ can be reimported and subsequently hydroxylated intracellularly by the monooxygenase PqsH to form PQS. Notably, the thioesterase PqsE, essential for pyocyanin production, is also involved in PQS synthesis or its regulatory circuit (Baldelli et al. 2020). PQS functions not only by binding its receptor PqsR to regulate virulence factors (e.g. elastase, lectin, and pyocyanin) but also by chelating Fe^3+^ to form a PQS-Fe^3+^ complex (Fig. 2C). This links QS directly to iron homeostasis and , through the regulation of efflux pump genes like *mexGHI-opmD*, to multidrug resistance (Cao et al. 2019).

Fatty acid derivatives: The diffusible signal factor (DSF) family comprises unsaturated fatty acid signals, first identified in *Xanthomonas* (Ryan et al. 2015). Their common structural feature is a *cis* carbon–carbon double bond, exemplified by DSF (*cis*-11-methyl-2-dodecenoic acid) and BDSF (*cis*-2-dodecenoic acid). DSF biosynthesis depends on the bacterial fatty acid synthesis (FAS) cycle and a key enzyme, RpfF, an enoyl-CoA hydratase. Carbohydrates and branched-chain amino acids can serve as precursors (Fig. 1F). Although *P. aeruginosa* lacks a canonical *rpf* cluster, it produces *cis*-2-decenoic acid, a DSF-family signal whose synthesis involves DspI, a homologue of RpfF (Soheili et al. 2015). This signal can be sensed by chemoreceptors containing a dCACHE domain (Zaman and Azam 2020).

Amino acid derivatives: This class illustrates how bacteria co-opt primary metabolic pathways for communication. 3,5-Dimethylpyrazin-2-ol (DPO) and AI-3 are both pyrazinone derivatives biosynthesized from l-threonine. In *V. cholerae* and enterohemorrhagic *E. coli*, l-threonine is oxidized by Tdh to 2-amino-3-ketobutyrate (AKB). AKB undergoes decarboxylation and condensation with l-alanine, eventually cyclizing to form DPO or AI-3 (Fig. 1D). AI-3 shares structural and functional similarities with host catecholamines like epinephrine and is sensed by the same two-component system (TCS; e.g. QseC/QseB), mediating cross-talk between intestinal pathogens and the host. Another amino acid-derived signal is cholera autoinducer-1 (CAI-1, (*S*)-3-hydroxytridecan-4-one), a long-chain aminoketone synthesized by the aminotransferase CqsA (Higgins et al. 2007, Wu et al. 2019). Due to its hydrophobicity, CAI-1 is often packaged into outer membrane vesicles for stable distribution in aqueous environments (Fig. 1C).

Cyclic Dipeptides (2,5-Diketopiperazines, DKPs): DKPs are stable cyclodipeptides and their derivatives widely produced by microorganisms, functioning as intra- and interspecific QS signals (Kim et al. 2021, Song et al. 2021). Their biosynthesis primarily occurs via two enzymatic routes: nonribosomal peptide synthetases and cyclodipeptide synthases (Zhang et al. 2019, Yee et al. 2023). For instance, in *S. baltica*, cyclo-(*L*-Pro-*L*-Phe) has been identified as a QSSM (Wang et al. 2019). This molecule binds to “orphan” receptor proteins LuxR01 and LuxR02 (which lack a cognate LuxI synthase). The resulting complexes regulate genes associated with spoilage potential (e.g. *speF*, involved in putrescine synthesis), biofilm formation (e.g. *pomA*), and trimethylamine production via the TorS/TorR system. DKPs also mediate interspecies communication, as seen between *Cronobacter sakazakii* and *B. cereus* (Bofinger et al. 2017).

Other emerging signaling molecules: The chemical repertoire of bacterial QSSMs continues to expand, encompassing structurally diverse molecules that regulate specialized behaviors. These include indole and its derivatives, which function as interspecific signals capable of reducing biofilm formation and virulence in *Listeria monocytogenes* (Rattanaphan et al. 2020); γ-butyrolactones produced by *Streptomyces* spp. to modulate bacterial secondary metabolite production (Liu et al. 2021); and dialkylresorcinols (DARs), identified as novel signaling molecules in the insect pathogen *Photorhabdus asymbiotica*, where they are synthesized via the DarABC pathway and perceived by the PauR receptor to regulate pathogenicity (Brameyer et al. 2015). Additionally, 3-hydroxy-palmitic acid methyl ester (3-OH-PAME) acts as a QSSM in *Ralstonia solanacearum*, modulating virulence factor expression (Achari and Ramesh 2015), while 2′-aminoacetophenone (2-AA) contributes to enhanced virulence and persistence in *P. aeruginosa* infections (B and yopadhaya et al. 2019).

In conclusion, the remarkable chemical diversity observed among bacterial QSSMs arises predominantly from the recruitment and modification of core metabolic pathways, including FAS, amino acid metabolism, and activated methyl cycles. This biosynthetic economy suggests an evolutionary adaptation favoring signal generation with minimal metabolic cost. The resulting specificity in intercellular communication is subsequently refined through precise structural elaboration of these core metabolites into unique signaling scaffolds, and further amplified by the parallel evolution of highly selective receptor and signal transduction systems. The fidelity and functional outcome of QS are thus governed by this intricate interplay between signal chemistry and receptor specificity. The following sections will systematically explore the molecular architecture, regulatory logic, and mechanistic diversity of these critical receptor and signaling pathways, which serve as the central apparatus for translating chemical population density into coordinated group behaviors.

## Receptors and signal transduction: the evolutionary and regulatory architecture of QS networks

The accurate detection of QSSMs and the subsequent reprogramming of cellular behavior are mediated by specialized receptor and signal transduction apparatus. These systems have diversified into a range of sophisticated yet frequently modular architectures, encompassing cytoplasmic transcription factors, membrane-bound sensor kinases, and intricate phosphorelay networks. Examination of these systems reveals recurring regulatory principles—including phosphorylation cascades, small RNA-mediated posttranscriptional control, and hierarchical signal integration—which collectively allow bacteria to effectively couple quorum-sensing information with other environmental and physiological inputs. The core regulatory mechanisms outlined in this section are summarized schematically in Fig. 2.

### Canonical receptor-transcription factor modules: LuxR-type and RRNPP Systems

The simplest and most widespread QS circuit involves a signal molecule directly modulating the activity of a transcriptional regulator, a process strictly governed by cell-density-dependent accumulation of signaling molecules to a critical threshold concentration.

### LuxI/LuxR paradigm

In the canonical AHL-based system of *V. fischeri*, the cytoplasmic transcription factor LuxR acts as both the receptor for 3-oxo-C6-HSL and the activator of the *luxCDABE* operon. This regulatory cascade is initiated only when the extracellular concentration of 3-oxo-C6-HSL reaches a specific threshold, typically in the nanomolar range (e.g. ∼10 nM) (Kareb and Aïder 2020, Patel et al. 2020). The LuxR-3-oxo-C6-HSL complex then binds to target DNA sequences, activating luciferase gene expression (Fig. 2A). This elegant, concentration-dependent, single-component logic is widely conserved in Gram-negative bacteria. For instance, *P. aeruginosa* employs two complete, hierarchically organized LuxI/LuxR-type systems (LasI/LasR and RhlI/RhlR) that govern virulence and biofilm formation. Each system responds to its cognate AHL—3-oxo-C12-HSL (synthesized by LasI) and C4-HSL (synthesized by RhlI)—at respective threshold concentrations, enabling integrated control of pathogenicity (Kumar et al. 2022, Li et al. 2022). Recent research further reveals nuanced regulatory mechanisms, such as in *Pseudoalteromonas*, where LuxR can modulate biofilm function independently of its cognate LuxI synthase, suggesting alternative activation pathways or integration of noncanonical signals (Nyffeler et al. 2022).

A significant expansion of this paradigm involves “orphan” LuxR homologs, which lack a cognate LuxI synthase. These “LuxR solos” can sense noncognate AHLs, exogenous signals from other species, or entirely different chemical classes, thereby broadening the bacterium’s environmental sensing capability (Bez et al. 2023). A notable mechanistic example is found in the food spoilage bacterium *S. baltica*. Its orphan receptors LuxR01 and LuxR02 bind the DKP signaling molecule cyclo-(*L*-Pro-*L*-Phe). When the concentration of this DKP reaches a functional threshold, the resulting complexes [LuxR01-cyclo-(*L*-Pro-*L*-Phe) and LuxR02-cyclo-(*L*-Pro-*L*-Phe)] regulate the expression of key genes such as *speF* (involved in putrescine synthesis), *pomA* (related to biofilm formation), and *torS* (a histidine kinase), collectively modulating spoilage potential and biofilm development (Fig. 2L) (Wang et al. 2019, Li et al. 2021). This illustrates how orphan receptors can co-opt non-AHL signals into canonical LuxR-type regulatory circuits, with activation strictly contingent upon signal accumulation to a requisite level.

### Intracellular peptide receptors: the RRNPP family

In Gram-positive bacteria, a major and mechanistically distinct class of QS utilizes cytosolic receptors belonging to the RRNPP family (named for Rap, Rgg, NprR, PlcR, and PrgX) (Hoover et al. 2015, Reuter et al. 2016). This system features a clear spatial and functional separation: small, modified AIPs are synthesized, processed, and secreted, then reimported into the cell upon reaching a threshold extracellular concentration—often in the low nanomolar range (e.g. ED_50_ ∼1–10 nM for *S. aureus* AIPs) (Mayville et al. 1999). The imported peptide directly binds to and activates its cognate intracellular transcription factor.

The NprR system in *B. cereus* provides a detailed mechanistic model. The precursor peptide pre-NprX is encoded by *nprX*, processed, and secreted. Its extracellular accumulation serves as a population density proxy. Once the concentration threshold is reached, NprX is imported via the Opp system. Inside the cell, binding of NprX to the receptor NprR induces a conformational change that promotes NprR tetramerization, converting it into an active transcriptional activator that induces expression of necrotrophic genes (Fig. 2G) (Zouhir et al. 2013). In the absence of the peptide ligand (i.e. at low cell density), NprR exists primarily as a dimer with potent phosphatase activity. This dimer dephosphorylates the sporulation phosphotransfer protein Spo0F, thereby repressing the phosphorelay leading to Spo0A activation and ultimately inhibiting sporulation (Yi et al. 2021, Rebuffat 2022). This ligand-dependent molecular switch—where the same protein (NprR) toggles between a phosphatase and a transcription factor based on the presence of a threshold concentration of its cognate peptide—represents a sophisticated form of signal integration and phenotypic fate determination.

Similar RRNPP-type mechanisms govern diverse processes in other *Firmicutes*, including competence development, bacteriocin production, virulence, and biofilm dynamics (Meijer et al. 2021). The specificity and sensitivity of these systems are finely tuned by the affinity between the peptide and its receptor (with dissociation constants often in the micromolar range, as seen in some AI-2 systems; Zhang et al. 2017), and by the efficiency of peptide import. However, the complete mechanistic details of peptide maturation, export, and the precise conformational changes in many RRNPP receptors remain active areas of investigation (Yi et al. 2021, Rebuffat 2022).

## TCS hubs: integrating QS with environmental sensing

Many QS circuits are built around TCS, which enable signal amplification and the integration of QS with diverse environmental stimuli through phosphorelay mechanisms. These systems typically consist of a membrane-bound sensor histidine kinase and a cytoplasmic response regulator.

### Membrane-bound histidine kinase receptors for peptide and small molecule sensing

A classic example is the *agr* system in *S. aureus*. The extracellular accumulation of AIP to a quantifiable threshold (typically 1–10 nM) is sensed by the membrane histidine kinase AgrC (Mayville et al. 1999). AIP binding triggers AgrC autophosphorylation, followed by phosphate transfer to the response regulator AgrA. Phosphorylated AgrA then binds to promoters P2 and P3, activating the transcription of RNA II (which includes the *agrBDCA* operon, forming a positive feedback loop) and RNA III (encoding virulence factors such as α-hemolysin and δ-toxin) (Fig. 2F) (Hoover et al. 2015, Reuter et al. 2016). This TCS-based architecture creates a strong autoinductive circuit that directly links population density to virulence expression.

*Staphylococcus aureus agr* system had been identified to be important in both intraspecies and interspecies interactions. With the stress of sublethal dose of antibiotics (streptomycin and tetracycline) frequently present in clinical environment and livestock, the enhanced *S. aureus* biofilm formation had been linked to upregulation of key genes in *agr* system. In the interspecies interaction of *S. aureus* with *Candida albicans*, which happens frequently in nosocomial sepsis and catheter-related bloodstream infections, *C. albicans* had been confirmed to augment *S. aureus* virulence via *agr* system and alpha-toxin-dependent mechanism. The major regulator of alpha-toxin production is governed by *agr* system dependent signaling. Modulation of extracellular pH to alkalinization could activate *agr* system in the interspecies coculture system. Mortality rates for *S. aureus* infections are ~10%, but can reach 80% when *C. albicans* is present, even with appropriate treatment. In addition, such changes in *agr* system had also been observed in the interspecies interaction of *S. aureus* with nonalbicans *Candida* species (*C. glabrata, C. dubliniensis, C. tropicalis, C. parapsilosis*, and *C. krusei*).

Similar to *agrBDCA* system in *S. aureus*, two homologous AIP-based QS systems *lamBDCA* and *lamKR* are acquired by *Lactiplantibacillus plantarum*. They cooperatively control adherence, cell morphology, and cell viability properties in *L. plantarum*. The two AIP-based QS systems both compose of histidine protein kinase (*lamC*/*LamK*) and response regulator (*lamA*/*lamR*). *LamA and lamR* regulates the downstream gene expression and phenotypes, including glass adherence and cell length. In addition, *lamBDCA* system has a precursor peptide encoding gene *lamD*, and *lamD* processing and posttranslational modification protein encoding gene *lamB*. During the interaction of *L. plantarum* with *Saccharomyces cerevisiae, lamBDCA* system had been identified to play an important role. *L. plantarum* and *S. cerevisiae* are frequently coidentified in natural fermentation system, including kefir, and sourdoughs. Upregulation of every gene in *lamBDCA* system leaded to the increased adhesion of *L. plantarum* and attached to *S. cerevisiae* cells. In the cross-kingdom interaction, cell density of *L. plantarum* must reach a certain threshold to induce the activation of *lamBDCA* system. *LamBDCA* system had also been reported to play a key role in the cross-kingdom interaction of *L. plantarum* with an opportunistic fungal pathogen *C. albicans. Lactiplantibacillus plantarum* and *C. albicans* are widely coexisted in human oral cavity and gastrointestinal tract. The application of *L. plantarum* as anti-*C. albicans* strategy has been explored in the past years and decades, but mostly focusing on *L. plantarum* metabolites including hydrogen peroxide, lactic acid, bacteriocin, biosurfactant, exopolysaccharides, and fatty acids. The importance and significance of QS system had been underestimated until the identification of lamBDCA system and AI-2 system of *L. plantarum* played a critical role in the inhibition of *L. plantarum* cells on *C. albicans* growth.

A distinct TCS hub senses small molecule signals that bridge bacterial communication and host physiology. In enterohemorrhagic *E. coli*, the histidine kinase QseC serves as a receptor for both the bacterial AI-3 and host catecholamines (epinephrine and norepinephrine). Upon binding, autophosphorylated QseC transfers phosphate groups to the response regulators QseB and QseF. Phosphorylated QseB activates the flagellar master regulator *flhDC*, modulating motility, while phosphorylated QseF regulates the expression of Shiga toxin genes (*stxAB*) (Cao et al. 2019). This system exemplifies how a bacterial TCS can integrate endogenous QS signals with host-derived chemical cues to coordinate virulence.

### Precision signal integration through multikinase phosphorelay networks

The most sophisticated implementations of threshold-based QS are found in *V*. species, which employ multi-kinase networks capable of integrating multiple autoinducer signals with weighted inputs. In *V. harveyi* and *V. cholerae*, three distinct signaling systems—CAI-1 (via CqsS), AI-2 (via LuxPQ), and in some species AHLs (via LuxN)—converge on a shared phosphorelay protein LuxU (Fig. 2D and H). Each sensor kinase exhibits a characteristic dissociation constant (Kd) for its cognate signal, establishing a unique concentration threshold for activation. For instance, the AI-2 receptor LuxP binds its boronated ligand with submicromolar affinity, while CqsS recognizes CAI-1 with nanomolar sensitivity (Zhang et al. 2020). This differential sensitivity allows the system to assign varying weights to different signals.

At the molecular level, the phosphorelay functions as a biochemical integrator. When autoinducer concentrations are below their respective thresholds, the sensor kinases act as net kinases, channeling phosphate through LuxU to the master regulator LuxO. Phosphorylated LuxO activates the transcription of small regulatory RNAs (Qrr1-4), which destabilize the mRNA of the master transcriptional activator LuxR/HapR, repressing collective behaviors. The phosphatase activity of each sensor is allosterically activated by ligand binding. Therefore, as the concentration of any autoinducer exceeds its threshold, the corresponding sensor switches from kinase to phosphatase dominance, initiating a net dephosphorylation of the LuxU–LuxO pathway. The system essentially computes a weighted sum of signal inputs; only when the combined signal strength (reflected in the net phosphorylation state of LuxO) crosses a critical threshold does the output switch from repression to activation of social behaviors (Dong et al. 2020, Sela et al. 2021). This design allows *Vibrio* to respond precisely to its social context, integrating both intraspecific signals (CAI-1) and interspecific cues (AI-2) into a coherent quorum decision.

### Threshold modulation by auxiliary regulators and environmental inputs

The concept of a fixed signal threshold is further refined by regulatory systems that dynamically modulate receptor sensitivity or signal availability. The GacS/GacA system in *Pseudomonas*, while its primary signal remains unidentified, exemplifies threshold modulation by nutritional status and other kinases. Gac is the acronym for global activator antimicrobial and cyanide synthesis. GacS is a membrane histidine kinase that can centralize multiple environmental stimuli and response regulation. At high cell densities, the sensor GacS is activated by as-yet-uncharacterized signaling molecules (Fig. 2E). The activated GacS is autophosphorylated and then activates the response regulator GacA. The expression of several small noncoding RNAs (sncRNAs) is upregulated by phosphorylated GacA. Subsequently, sncRNAs sequester the regulatory protein RsmA, and the activation of GacS inhibits the effects of these RsmA proteins. The RsmA proteins are RNA-binding proteins that can modulate the translation and the stability of target messenger RNAs. It is reported that GacS/GacA can promote the synthesis of AHLs in *Pseudomonas* (Anderson et al. 2017).

In *Pseudomonas, LadS* (loss of adherence sensor) and *RetS* (regulator of exopolysaccharide and type Ⅲ secretion system) are orphan sensor kinases without response regulators. *LadS* and *RetS* reportedly modulate the activity of GacS (Chambonnier et al. 2016). *LadS* enhances the activity of GacS by transferring additional phosphate groups to the Hpt domain of GacS. *RetS* inhibits the activity of GacS through three different mechanisms. First, RetS prevents the transmission of phosphate groups to GacA by capturing the phosphate groups from the HK domain of GacS. Second, RetS catalyzes the dephosphorylation of GacS. Third, *RetS* binds tightly to the HK domain of GacS, which hinders the autophosphorylation of GacS. Finally, there are other membrane histidine kinases (e.g. PA1611) that can also affect the activity of GacS (Kong et al. 2013).

Similar threshold modulation occurs in AI-2 signaling in *E. coli* and *Salmonella*. In *E. coli*, the *lsr* operon, regulated by *LuxS*, comprises *lsrA, lsrC, lsrB, lsrF*, and *lsrG* (Fig. 2I). The cytoplasmic protein *LsrB*, encoded by the *lsrB* gene, can bind to non-borated AI-2, *R*-2-methyl-2,3,3,4-tetrahydroxytetrahydrofuran (*R*-THMF). The *lsr*K *and lsr*R genes are located upstream of the *lsr* operon. The lsrK-encoded protein is a kinase, and the protein LsrR, which is encoded by the *lsr*R gene, can inhibit the transcription of the operon *lsr*. The content of extracellular AI-2 is less in its early growth stage. After reaching the threshold density, AI-2 binds to the protein LsrB (with a binding affinity (Kd) in the range of 0.19–0.81 μM) and is transported into the cell by the *lsr* transport system (Torcato et al. 2019). Then, AI-2 molecules enter the cell and are phosphorylated by LsrK to form phosphate-AI-2 (P-AI-2). The P-AI-2 combines the protein LsrR to relieve the inhibitory effect of LsrR on the *lsr* operon. Finally, the transport system Lsr is further induced to increase intracellular AI-2. Moreover, the enzyme proteins LsrF (a coenzyme A-dependent thiolase) and LsrG (a cofactor-independent monooxygenase) process P-AI-2, regulating intracellular AI-2. Ultimately, AI-2 governs the process of bacterial cell internalization, phosphorylation, and depletion.

While the synthetic pathways of AI-2 are conserved across different bacterial species, variations exist in the mechanisms of AI-2 recognition and transmission. For example, in the cross-kingdom interaction between *L. plantarum* and *C. albican*, key gene in AI-2 QS system, *luxS* gene, was significantly downregulated when the cell density of *L. plantarum* reached a threshold. Downstream genes involved in the AI-2 pathway, including *metE, metH*, and *metK*, were also downregulated. While in interaction of *L. plantarum* with *E. coli* O157:H7, *L. monocytogenes*, and *S. aureus* (Xin et al. 2002, Dong et al. 2020), *luxS* gene had been shown to be significantly upregulated. The diverse change in AI-2 system in different dual-species interactions are interesting and its mechanism is remained to be explored.

In summary, TCS hubs in QS are far more than simple on-off switches. They are sophisticated signal-processing units that establish precise concentration thresholds for activation, often through high-affinity receptor–ligand interactions. These thresholds can be static, defined by biophysical constants, or dynamic, modulated by auxiliary regulators, transport systems, and environmental conditions. The ability to integrate multiple signals with different thresholds—as seen in the *Vibrio* phosphorelay—allows bacteria to make nuanced, population-level decisions that integrate information about self-density, other species, and host status, ultimately optimizing the timing and investment in social behaviors such as virulence, biofilm formation, and AMR.

### Secondary messenger integration: c-di-GMP as a central QS effector

The ubiquitous bacterial second messenger cyclic diguanylate (c-di-GMP) frequently serves as a key downstream effector of QS, directly linking cell–cell communication to lifestyle switches between motility and biofilm formation (Valentini and Filloux 2019).

DSF systems: In *Xanthomonas*, the regulation mode of DSF involves a TCS (RpfC/RpfG) (Ling et al. 2019). The sensor kinase RpfC is inactive when the concentration of extracellular DSF is low. Inactive RpfC can bind the protein RpfF through the REC domain and inhibit the synthesis of DSF signaling molecules (Zhou et al. 2017). The global regulatory factor Clp binds to the secondary messenger c-di-GMP (Wang et al. 2018). The complex (c-di-GMP and Clp) binds to the promoter region of the rpfB gene and inhibits its transcription (Wan et al. 2021). Additionally, Clp, when combined with c-di-GMP, is unable to bind to the promoter of the pathogenic gene *eng*XCA (Fan et al. 2022). When the extracellular DSF concentration reaches the threshold (typically peaking in the range of 1–10 μM during late exponential phase), RpfC receptors respond to DSF molecules (Deng et al. 2011). Autophosphorylation activates RpfC, which phosphorylates RpfG (Wang et al. 2022). The phosphorylated RpfG exhibits phosphodiesterase activity, degrading c-di-GMP to guanylate (GMP). The Clp that does not bind to c-di-GMP will fall off from the *rpf*B promoter region so that *rpf*B can be transcribed. The gene *rpf*B encodes a fatty acyl-coenzyme A ligase RpfB that may participate in the degradation of DSFs (Huedo et al. 2014). Free Clp can tightly bind to the promoter region of the gene *eng*XCA, initiating the expression of pathogenic factors (Fig. 2J).

In *Burkholderia*, a different regulatory mode exists, primarily reported in *B. cepacian* and *B. cenocepacia* (Huedo et al. 2019). Instead of RpfC/RpfG system, RpfR (a protein with PAS, GGDEF, and EAL domains) is responsible for signal sensing (Abaturov and Kryuchko 2019). The GGDEF domain is related to the synthesis of c-di-GMP (Hermanas et al. 2021). Furthermore, the EAL domain is responsible for the degradation of c-di-GMP (Dong et al. 2022). In *B. cenocepacia*, RprF is involved in the synthesis of the signaling molecule BDSF (Boon et al. 2008). When the concentration of extracellular BDSF is low, RpfR protein is bound to RpfF protein to inhibit the thioesterase activity of RpfF (Dow et al. 2016). The concentration of extracellular BDSF reaches the threshold with the expansion of the cell population (Wang et al. 2022). It is then is perceived by RpfR and bound to the PAS domain of RpfR (Tian et al. 2022). The BDSF–RpfR complex degrades c-di-GMP, which causes a decrease in the intracellular c-di-GMP concentration (Jung et al. 2017). Although RpfC/RpfG TCS and RpfR are two different ways of perception, they transmit DSFs to the secondary messenger c-di-GMP. The c-di-GMP interacts with subsequent regulatory factors and plays a regulatory role at the transcriptional and translation levels (Fig. 2K) (Huang et al. 2020). A high concentration of c-di-GMP is conducive to the formation of bacterial biofilm and vice versa, a low concentration limits the production of virulence factors (Valentini and Filloux 2019).

PQS system: In *P. aeruginosa*, the PQS system also intersects with c-di-GMP-mediated regulation. The PQS system is indispensable for bacterial material transportation and the production of virulence factors (Cao et al. 2019). The complex PqsR–PQS regulates the expression of efflux pump genes such as *mexGHI* and *ompD*. The MexGHI-OpmD efflux pump can excrete antimicrobials with structural similarity to PQS, contributing to drug resistance (Cao et al. 2019). This efflux activity and the associated stress response are linked to broader physiological adaptations, including those influenced by cellular c-di-GMP levels, which affect biofilm architecture and virulence (Cao et al. 2019, Valentini and Filloux 2019).

### Hierarchical cross-talk and network integration

QS systems within a bacterium are often organized hierarchically and engage in extensive cross-talk. The classic example is the Las-Rhl-PQS hierarchy in *P. aeruginosa* (Higgins et al. 2018). The Las system (LasI/LasR, 3-oxo-C12-HSL) activates the expression of the rhlI/R genes and the pqsR gene. The Rhl system (RhlI/RhlR, C4-HSL) is partially subordinate to Las and provides negative feedback on the PQS system (pqsABCDE and pqsR) (Higgins et al. 2018). The PQS system, in turn, produces signals that further regulate virulence factors and interact with the other two systems (Higgins et al. 2018, Cao et al. 2019). This interconnected network allows for temporal gene expression programs and fine-tuned control over a large virulence regulon, integrating signals that also modulate secondary messenger pathways like c-di-GMP.

The recurring theme of diverse QS systems—whether employing AHLs, DSF, AQs, or peptides—converging on the modulation of c-di-GMP highlights a conserved mechanism for translating population-sensing information into tangible phenotypic outputs related to adhesion, virulence, and community persistence (Wang et al. 2018, 2022, Valentini and Filloux 2019).

## Other QS systems in the mechanistic attitudes of bacterial infections

The above-discussed QS systems are documented mainly in pathogenic microbes. Nonetheless, numerous QS insights are addressed for rational outcomes in pathogenesis and virulence mechanisms. For example, indole and its derivatives are a new type of interspecific signaling molecules, which can reduce the biofilm formation and virulence of *L. monocytogenes* (Rattanaphan et al. 2020). AIPs derived from *L. monocytogenes* and their analogues have been established for *N*-methylation or thioester-to-thioether substitutions to regulate signal transmission (Bejder et al. 2024). A novel efflux pump encoded by *emrE* is envisioned to confer higher tolerance levels against quaternary ammonium sanitizers, thereby enhancing the prospects for food industrial stress resistance (Kovacevic et al. 2016). *Listeria monocytogenes* establishes several mechanisms, including the Lux system, Agr system, EPS generation, stress, and adaptive responses for QS systems. Furthermore, *PrfA* gene expression profiles are necessary for survival in harsh settings and with food preservatives/disinfectants and are linked to AMR patterns. Furthermore, host–pathogen interactions and regulation are correlated to patterns of cross-domain and interspecies interactions (Barber et al. 2022). The agr system has also been confronted with heterogeneous expression and autoinduction strategies, ranging from single-cell to population intensification in *L. monocytogenes* (Garmyn et al. 2011). Furthermore, the Agr-D QS system was identified as a global regulator of gene expression contributing to biofilm formation and subsequent invasion and virulence in the saprophytic and parasitic behaviors of *L. monocytogenes* (Riedel et al. 2009). The *L. monocytogenes* QSSMs are aggravated for virulence, pathogenesis, and AMR patterns (Meireles et al. 2024). The PrfA QS system in *L. monocytogenes* accounts for the cascade of events in transcriptional activation and virulence gene expression encompassing biofilm regulation (Luo et al. 2013). The VirSR–VirAB two-component regulatory system has been shown to influence biofilm adhesion properties, but not swarming motility, in *L. monocytogenes*, as related to food safety protocols (Guo et al. 2023). Thus, the listeriosis-causing pathogen and food security condemning QS systems and regulatory patterns are vividly discussed for pattern variations. Similarly, the unusual occurrence of γ-butyrolactone from *Streptomyces* spp. can regulate the production of bacterial secondary metabolites (Liu et al. 2021). However, 2’-aminoacetophenone (2-AA) acts as a QSSM, contributing to the increased virulence of *P. aeruginosa* and persistence, which accounts for the escalation of recurrent infections (Bandyopadhaya et al. 2019).

Recently, DARs, as novel and extensive signaling molecules, have been shown to regulate the pathogenicity of the insect pathogen *P. asymbiotica* (Brameyer et al. 2015). Many studies have confirmed that photopyrones are involved in the synthesis of different natural products in *P. luminescens* (Kresovic et al. 2015). *Photorhabdus* species possess LuxR solos, mainly PAS4-LuxR solos for symbiosis and pathogenesis for QS-based interkingdom signaling mechanisms (Brameyer et al. 2014). Similarly, *P. luminescens* and *P. temperate* utilize the LuxR–LuxI system, which senses α-pyrones (photopyrones produced by pyrone synthase) through the PluR receptor. PluR homolog designated as PauR, senses DARs involved in the DarABC pathway in *P. asymbiotica* QS systems collaging pathogenic profiles (Brameyer and Heermann 2017). The KdpD/KdpE TCS in *P. asymbiotica* revealed potassium-sensing-mediated *M. sexta* hemocytes phagocytosis through proteases and neutrophil killing mechanism (Freeman et al. 2013).

Other QS systems that are distinct have been reported in *R. solanacearum*, where 3-hydroxy-palmitic acid methyl ester (3-OH-PAME) serves as a QS signaling molecule modulating the expression of virulence factors (Achari and Ramesh 2015). Unusual QS systems documented include the *R. solanacearum* species complex (RSSC), comprising *phc* QS encoded by the *phc*BSRQ operon and the *phcA* gene. Methyl 3-hydroxymyristate (3-OH MAME) or methyl 3-hydroxypalmitate (3-OH PAME) acts as QSSMs for virulence regulation in RSSC (Kai 2023). The RasI/R QS systems (PhcBSR and SolI/R) have been cataloged for their similarity to LuxI/LuxR homology, specifically in the synthesis of *N*-(3-hydroxydodecanoyl)-homoserine lactone (3-OH-C12-HSL), which is associated with phytopathogenicity (Yan et al. 2022). QSSMs involving 3-OH MAME or 3-OH PAME synthesized by PhcB methyltransferases in *phc*QS systems in RSSC virulence (Ujita et al. 2019). The RSSC comprises EPS I and ralstonin A, along with *phc* QS system for pathogenicity against *Fusarium oxysporum* Chlamydospores (Tsumori et al. 2023). The 3-OH PAME acts as a diffusible signal regulating *phc* QS system evolution in *R. solanacearum* (Kai et al. 2015). Ralsolamycin mediates inter-kingdom signaling system by PhcBSR QS systems in *R. solanacearum* (Li et al. 2017). Apart from the above, 2-hydroxy-4-((methylamino)(phenyl)methyl) cyclopentanone was also confirmed for QS regulation by *R. solanacearum* (Kumar et al. 2016). The *trp*EG gene synthesis of anthranilic acid demonstrated inter-kingdom QS regulation (Song et al. 2020). Sequence of events in enhanced pathogenicity shows PhcK gene mediating PhcA regulation of the *R. solanacearum* strain OE1-1 QS system (Senuma et al. 2020). Further, the 2',3'-cyclic guanosine monophosphate (2',3'-cGMP) signals also regulate virulence and physiology (Li et al. 2023). The PrhX adjacent to the PhcB–PhcA QS system accounts for the type III secretion system (Huang et al. 2024). Thus, the QS systems, which are not confined to specific regulatory mechanisms and additional machinery, are discussed in the context of disease resistance management. Furthermore, QS systems and precise modalities for pathogenicity, polymicrobial interactions, and AMR require broader insights for future research.

### Significance of QS systems in pathogenicity, polymicrobial interactions, or resistance

QS systems in pathogenicity are well-documented in numerous reports, which address AI-3 molecules and their analogues against various Gram-positive and Gram-negative bacterial pathogens (Kim et al. 2020). Fur and SmcR coordinately regulate the iron concentration apart from *vvsAB* required for vulnibactin synthesis in *Vibrio vulnificus* virulence and pathogenicity (Wen et al. 2012). Immune system-mediated QS regulation in cardiovascular diseases incited by *S. aureus* biofilms accounts for increased pathogenesis and invasion mechanisms (Taj and Chattopadhyay 2004). *P. aeruginosa* contains a two-component QS system consisting of the sensor histidine kinase GacS (LemA) and the highly conserved response regulator GacA, crucial for bacterial physiology and pathogenesis (Adamiak et al. 2025). Community-associated methicillin-resistant *S. aureus* (CA-MRSA) involves the mec and agr QS systems, which are interdependent in terms of pathogenesis and persistence (Cheung et al. 2011). Furthermore, QS systems are associated with multidrug resistance in *P. aeruginosa* (Maiga et al. 2025). QS-mediated efflux mechanisms contribute to AMR patterns and corroborate the use of efflux pump inhibitors for managing pathogenesis in chronic and recurrent infections (Sionov and Steinberg 2022). Thus, pathogenesis in pathogenic bacteria is coerced by the significance of QS systems. However, polymicrobial interactions complicate the complexity of QS in AMR patterns, highlighting the critical severity and dire attention required.

Polymicrobial interactions are commonly encountered in adverse conditions, such as cystic fibrosis, involving *P. aeruginosa* and other pathogens (Luján et al. 2022). Microbial community interactions between QS systems involve diffusible signals resulting in ecosystem dynamics (Abisado et al. 2018). The LasR/LasI, MvfR/PqsABCDE, and RhlR/RhlI QS systems in *P. aeruginosa* are regulated by MvfR for facilitating polymicrobial interactions and recurrent infections in ESKAPE pathogens (Wheeler et al. 2025). Quinolinones proved effective polymicrobial interactions targeting PQS systems in *P. aeruginosa* and *S. aureus* using tobramycin (Murray et al. 2022). Polymicrobial interactions and inhibition of QS systems are addressed to prevent chronic wound infections, otitis media, dental caries, and cystic fibrosis (Anju et al. 2022).

*P. aeruginosa*, along with *Staphylococcus, Acinetobacter, Klebsiella, Enterococcus*, and *Candida*, are among the deleterious polymicrobial pathogens in humans (Sachdeva et al. 2025). Future research on persistent *P. aeruginosa* infections requires a comprehensive understanding of the physiology of chronic diseases by polymicrobial communities that are resistant to antibiotic therapy (Bisht et al. 2020). The indirect pathogenicity of *Haemophilus influenzae* and *Moraxella catarrhalis* in polymicrobial otitis media transpires through an interspecies QS mechanism. Effective vaccination against unencapsulated *H. influenzae* strains through airway infections may substantially influence chronic *M. catarrhalis* disease by eliminating a reservoir of the AI-2 signal that facilitates *M. catarrhalis* persistence within biofilm (Armbruster et al. 2010). AMR in polymicrobial biofilm communities encompasses interspecies transfer of antibiotic resistance genes (ARGs), β-lactamase-mediated antibiotic inactivation, alterations in gene expression prompted by metabolites and QS signals, disruption of the electron transport chain, and modifications in cell membrane properties (Orazi and O’Toole 2019). Polymicrobial infection patterns caused by *P. aeruginosa* suggest that therapeutic techniques targeting Gram-positive bacteria may effectively mitigate the severity of *P. aeruginosa* polymicrobial infections (Korgaonkar et al. 2013). Polymicrobial interactions of *L. monocytogenes* have been shown to involve EPS synthesis-associated genes regulated by the agr QS system, as well as with other pathogens, such as *S. enterica* (Banerji et al. 2022). Nevertheless, AMR patterns, pathogenicity, and polymicrobial interactions will pave the way for effective QS inhibition.

AMR mechanisms and associated QS systems involve QSSMs, as well as several cellular processes, including virulence and drug resistance mechanisms, that contribute to antibiotic tolerance (Zhao et al. 2020). Recent research in AMR has envisioned the use of QSSMs inhibition without the use of antibiotics, targeting AHLs, furanosyl borate, hydroxyl-palmitic acid methyl ester, and methyl-dodecanoic acid, along with other components such as furanones, glycosylated chemicals, heavy metals, and nanomaterials (Haque et al. 2021). On the contrary, the MexAB–OprM efflux system in *P. aeruginosa* did not establish AMR due to QS systems, but rather by the *las* and *rhl* QS systems (Bratu et al. 2006). *Agr* QS systems in *S. aureus* and *S. epidermidis* infections confer virulence and AMR (Singh and Ray 2014). Complex QS interplay and antibiotic resistance are corroborated in *P. aeruginosa*, emphasizing the role of QQ in combating AMR (Sikdar and Elias 2022). QS systems attributing AMR also include autoinducer synthase, transcriptional regulator, bacterial capsule, iron acquisition, adherence factors, lipopolysaccharide, poly-*N*-acetylglucosamine (PNAG) biosynthesis, and motility in ESKAPE pathogens, contributing to their virulence (Santajit et al. 2022). Furthermore, recent research suggests targeting QS inhibitors against QSSMs without the use of broad-spectrum antibiotics to combat AMR.

QS inhibitors are proposed as an effective alternative in combating MDR pathogens that target LuxR–AHL receptors, which are histidine kinase receptors present in Gram-negative bacteria. The family of gliptins, comprising sitagliptin, was found effective against *S. aureus* and *P. aeruginosa* (Khayat et al. 2022). QQ and QS inhibition target ARGs in the treatment of chronic illnesses and polymicrobial interactions, such as microbiome infestation and antibiotic resistance (Tripathi et al. 2022). QSSMs and signal transduction molecules involved in QS and biofilms are innovatively addressed to achieve rational clinical outcomes, thereby bypassing the need for antibiotic usage (Juszczuk-Kubiak 2024). QS systems in polymicrobial interactions facilitate the production of AHLs and AIPs, as well as inhibitors such as furanones and AHL analogs, for the prevention of AMR and QS inhibition (Cui and Kim 2024). AHL-based QS systems in *P. aeruginosa* and *B. cepacian* complex were confined to the quorum-sensing inhibitors (QSIs), baicalin hydrate, cinnamaldehyde, and hamamelitannin against *S. aureus, in vitro* and *in vivo* (Brackman et al. 2011). QSIs interact with SdiA and LsrR proteins, increasing resistance mechanisms due to high toxicity (Li et al. 2021). Evolution of QSIs has been a phenomenon in AMR, and antibiotic research employing QS systems also results in multiquorum-sensing inhibitor-resistant bacteria (Koul et al. 2016). Nonantibiotic combinations have been stressed for QS inhibitors for almost all AMR pathogens (Tonkin et al. 2021). Moreover, drug combinations with QSIs have been proven effective against QS systems in *P. aeruginosa*, like *N*-(2-pyrimidyl) butanamide (Furiga et al. 2015) and *N*-decanoyl-l-homoserine benzyl ester (Yang et al. 2012). Hence, the scenario of QS inhibition and arrest of polymicrobial infections is gaining momentum in recent years.

The significance of QS systems lies in their involvement in antibiotic resistance and in polymicrobial infections. These include device-associated infections, persistent wound infections, and lung infections in cystic fibrosis patients. This significance explains the growing interest in promising QSIs. The inherent mechanisms of lowering virulence and inhibiting biofilm development, as observed in laboratory and clinical investigations involving furanones and AHL analogues, provide insight into the global scenario of antibiotic resistance in polymicrobial infections. Thus, the research points to the development of therapeutic approaches that disrupt bacterial communication and enhance antibiotic efficacy, thereby linking QS to antibiotic resistance (Cui and Kim 2024). Since QS is linked to virulence and the production of biofilms, QQ inhibition of microbial illnesses, including biofilms formed by pathogenic bacteria, confronts the antibiotic resistance patterns (Juszczuk-Kubiak 2024). Furthermore, QS mechanisms have been implicated in the pathogenesis of molecular diseases, including the suppression of virulence and defense, as well as the accumulation of infectious roles and AMR (Deep et al. 2011). *S. aureus* QS systems involve a two-component QS system encoded by the *agr* locus (Rutherford and Bassler 2012). Two complete circuits involving AHL signals and a third system utilising quinolone signals constitute *the P. aeruginosa* QS system. The expression of genes that code for virulence factors, regulated by the above three QS circuits, contributes to cumulative regulatory benefits (Zhao et al. 2020). The current QQ research corresponds to novel strategies of bacterial communication for biomedical applications. Furthermore, QSI offers novel AMR mechanisms and effective delivery operations. Combatting antibiotic resistance by exploring the promise of QQ in targeting bacterial virulence is gaining momentum (Patel et al. 2025).

The harmful Gram-positive and Gram-negative bacterial pathogenicity has been attributed to carbohydrates and associated virulence genes (Suresh et al. 2021). QSIs are collated to Furanones, glycosylated chemicals, heavy metals, and nanomaterials for AMR patterns due to QS systems (Haque et al. 2021). Similarly, lung epithelial infections and MvfR inhibition modify competitive interspecies interactions and maintain *P. aeruginosa*’s cohabitation with the ESKAPEE pathogens under investigation. MvfR inhibition attenuates virulence and competitive interactions across several species. The influence of the antivirulence strategy in microbial ecology, significance for treating polymicrobial infections and complexity addresses the significance of QS in interspecies interactions (Wheeler et al. 2025). Moreover, Hfq-binding small RNA PqsS was observed for the regulation of *P. aeruginosa pqs* quorum sensing-mediated virulence (Jia et al. 2024). The QS network and regulatory mechanisms are correlated to oral biofilm microbiota using *the in vitro* oral biofilm microbiota assembling model, showing a theoretical foundation for the natural assembly of complex microbiota (Su et al. 2023). The recent research indicates nanomaterials as potential regulators of bacterial QS systems and optimisation (Hu et al. 2024). Hence, the significance of QS systems in pathogenicity, polymicrobial interactions, and/or AMR is redressed for further applications.

### Therapeutic and biotechnological applications of targeting QS systems

Targeting bacterial QS represents a fundamental shift in strategy, moving from traditional bactericidal approaches to antivirulence therapy. The goal is not to directly kill pathogens but to “disarm” them by disrupting cell-to-cell communication. This effectively reduces virulence factor production, inhibits biofilm formation, and minimizes host tissue damage, while avoiding the lethal selective pressure that drives antibiotic resistance (Rasmussen and Givskov 2006, Dal et al. 2025). This approach lowers pathogenicity and allows the host immune system to clear infections more efficiently, as demonstrated in *P. aeruginosa* keratitis models, where the QS inhibitor furanone C-30 improved clinical outcomes without reducing bacterial load (Dal et al. 2025). Beyond its therapeutic potential, QS and QQ principles have been successfully applied across diagnostics, synthetic biology, industrial processes, and environmental management, highlighting their versatility as both therapeutic targets and bioengineering tools (Mangwani et al. 2012, Abbamondi and Tommonaro 2022).

A broad spectrum of QSIs has been identified, including natural compounds (curcumin, quercetin, baicalein, allicin, and cinnamaldehyde) and synthetic derivatives (furanone C-30 and halogenated furanones). These agents inhibit QS-regulated virulence in pathogens like *P. aeruginosa* by competitively binding to receptors (e.g. LasR), downregulating QS gene expression, and reducing AHL production (Packiavathy et al. 2014, Ouyang et al. 2016, Luo et al. 2017). This antivirulence approach shows promise against multidrug-resistant pathogens including *Vibrio* spp., *E. coli*, and *A. baumannii* (Shaaban et al. 2019, Patel et al. 2023).

QSIs are increasingly studied in combination with conventional antibiotics to enhance efficacy through biofilm disruption, efflux pump inhibition, and antibiotic susceptibility restoration. Cinnamaldehyde combined with tobramycin improves biofilm eradication, while curcumin reduces the minimum inhibitory concentration of azithromycin and gentamicin against *P. aeruginosa* (Bahari et al. 2017, Topa et al. 2020). Such combinations improve outcomes in chronic infections while mitigating resistance development by reducing selective pressure (Kalia et al. 2014). Clinical relevance is supported by studies showing QQ enzymes like *Sso* Pox-W263I lactonase significantly enhance the susceptibility of clinical strains to antibiotic (Mion et al. 2019).

QS signaling molecules serve as early, specific infection biomarkers. For instance, detecting AHLs (e.g. 3O-C12-HSL) in cystic fibrosis patient sputum can indicate *P. aeruginosa* infection progression. This principle enables microbial biosensor development—genetically engineered strains like *E. coli* qSB401 and *Chromobacterium violaceum* CV026 produce detectable signals in response to specific QS molecules, enabling sensitive, pathogen-specific diagnostics (Winson et al. 1998, Dimitrova et al. 2023).

QS circuits enable programmable, population-density-dependent genetic systems. Applications include engineered biosensors for environmental monitoring, controlled biochemical synthesis, and mixed-species fermentations (Mangwani et al. 2012). Therapeutic delivery systems like synchronized lysis circuits enable density-dependent therapeutic release in tumor microenvironments, while orthogonal QS systems coordinate engineered probiotic consortia for combination therapies (Chowdhury et al. 2019, Guo et al. 2025).

QQ strategies combat biofouling across sectors. Encapsulated QQ bacteria (e.g. *A. pittii* HITSZ001) in microbial fuel cells control biofouling in membrane bioreactors, enhancing wastewater treatment stability while facilitating energy recovery (Wang et al. 2023b). QQ enzymes immobilized on nanofiltration membranes significantly reduce biofilm formation (Yeon et al. 2009, Yu et al. 2014). QQ-based coatings prevent fouling on ship hulls and medical devices like urinary catheters (Ivanova et al. 2015, Riga et al. 2017). In aquaculture, QQ probiotics protect fish from pathogens like *A. hydrophila* and *Vibrio* spp., reducing disease mortality and antibiotic use (Cao et al. 2012, Vinoj et al. 2014). QS regulates industrial and ecological processes in specialized environments. In acidophilic *Acidithiobacillus*, the LuxI/R-like AfeI/R QS system regulates metabolism relevant to bioleaching, offering potential for optimizing metal recovery and mitigating acid mine drainage (Gao et al. 2021). Understanding QS in oral biofilms and bacterial-fungal interactions (e.g. via LuxR solos in *Pandoraea* species) provides theoretical foundations for manipulating complex microbiota (Chua et al. 2019, Nagi et al. 2023).

The applications of targeting QS systems extend beyond antimicrobial strategies to encompass synergistic therapies, advanced diagnostics, and versatile biotechnological tools—enabling solutions from biofilm control and industrial process enhancement to synthetic microbial community programming. This broad relevance across healthcare, industry, and environmental sustainability underscores QS systems’ importance. Future progress depends on translating these concepts into practical solutions for persistent challenges like AMR, chronic infections, and biofilm-related problems in diverse settings.

## Highlights, challenges, and future perspectives

Since the discovery of QS systems in 1970, structurally diverse signaling molecules and several QS systems interrogating cell–cell communication have been uncovered. Meanwhile, increasing evidence shows that almost all the adaptability of environmental microorganisms and the pathogenicity of multiple drug-resistant pathogens are related to QS systems. In this review, we summarized all reported bacterial QS systems and QS-regulated signaling molecules to the best of our knowledge. However, bacterial QS systems are complex, and the present studies cannot fully and perfectly cover them. Furthermore, the bacteria we found and cultured represent only a small part of those in nature, and the QS systems that have been studied likely represent only a small percentage of those yet to be identified. The identification diverse and discovery of new QS systems and signaling molecules still need to be carried out continuously.

Apart from bacterial resistance, antimicrobial drug misuse/abuse, and intercontinental travel-triggered resistance lead to life-threatening resistance mechanisms (Pulingam et al. 2022). The specific AMR mechanisms in *Burkholderia* species have been reported to contribute to species-specific virulence and pathogenicity. Factorial interplay studies on pathogen-specific QS systems are necessary (Rhodes and Schweizer 2016). Biofilm formation, bioluminescence, virulence, pathogenic profiles, and AMR are the inherent mechanisms of expression. Furthermore, the diversity of signaling molecules comprises a variety of autoinducer molecules. AI-2 and AHLs are responsible for interspecific and intraspecific bacterial cell–cell communication. Environmental adaptive strategies mediated by QS include nutritional acclimatization, as well as temperature, pH, and host-related survival mechanisms. The diverse and complex QS mechanisms involved in disease resistance also require assessment of cross-kingdom interactions. Bacterial cross-talk and interfering signaling cascade interactomes necessitate the appropriate elicitation of signaling. Modification of signaling cascades in Gram-negative and Gram-positive bacteria, as well as the interplay between these and gene regulation, will provide ample future insights. The specificity and sensitivity of QS signaling cascades in bacterial communities will derive more platforms for holistic research prospects.

In this review, the reported QS systems and signaling molecules in bacteria up to now are compiled to the best of our knowledge. The evolutionary significance of QS systems is reported to the effective abatement of multidrug and disease resistance. The present report provides favorable support for the involvement of CAI-1 and associated signaling molecules, such as AI-2. Thus, the concepts of QS need to be revisited to find new signaling molecules and necessary gene/protein regulatory networks. Interestingly, many bacteria contain orphan proteins that lack known corresponding signaling molecule synthases. QS systems include the TCS-based QS systems, and several inclusive mechanisms were addressed in the present review, for one thing, as QS interference, QS inhibition, and QQ will aid in comprehensive future research. Recently, antimicrobial peptides have been cataloged for combating drug resistance (Xuan et al. 2023). Further antipersistent mechanisms are required for arresting persistent and recurrent bacterial infections (Kaushik et al. 2022). Mitigation of bacterial biofilms and drug resistance requires additional research, including environmental behaviors, fluid medium, and surface topology, as well as immune mechanisms (Mukherjee and Bassler 2019).

The present review summarizes the QS signaling molecules and their necessary regulatory interactions. Various Gram-negative and Gram-positive QS systems and associated interspecific cell–cell communication are critically discussed. However, future research in coercing various QS systems requires a multifaceted approach. QS system-based research and its latest strategies have been addressed in recent years, and successful clinical studies reveal future commercialization (Ye and Chen 2022, Darby et al. 2023, Zeng et al. 2023). Biofilm inhibition and multidrug-resistance-based QS systems are compiled, eluding environmental applications like sewage treatment. Apart from multidrug resistance and pathogen biology, QQ needs to be unraveled for resistance, interference, gene/protein interactions, and regulatory mechanisms. However, several research questions need to be answered to gain a comprehensive understanding of disease resistance mediated by QS systems, including the discovery of new signal molecules that regulate QS. Furthermore, additional QQ mechanisms, metabolomics, systems biology, the CRISPR/Cas system, and culturomics should be emphasized for successful outcomes. It is expected that more QS systems and novel signaling molecules in bacterial cell–cell communications will be elucidated and identified in the future. These findings will provide new strategies for combating multidrug-resistant bacterial infections. Moreover, the repurposing of combination therapy that employs QSIs targeting QS complexities is aimed at either overcoming AMR patterns or enhancing therapy with rational QSIs for broad-spectrum activities.

## Data Availability

This manuscript does not generate any code or data.

## References

[bib1] Abaturov AE, Kryuchko TE. Pharmacological effect on biofilm dispersion. Derivatives of the diffusible signal factor family. Zdorovʹe Rebenka. 2019;14:386–92.

[bib2] Abbamondi GR, Tommonaro G. Research progress and hopeful strategies of application of quorum sensing in food, agriculture and nanomedicine. Microorganisms. 2022;10:1192. 10.3390/microorganisms10061192. 35744710 PMC9229978

[bib3] Abisado RG, Benomar S, Klaus JR et al. Bacterial quorum sensing and microbial community interactions. mBio. 2018;9:e02331–17. 29789364 10.1128/mBio.02331-17 PMC5964356

[bib4] Achari GA, Ramesh R. Characterization of bacteria degrading 3-hydroxy palmitic acid methyl ester (3OH-PAME), a quorum sensing molecule of *Ralstonia solanacearum*. Lett Appl Microbiol. 2015;60:447–55. 10.1111/lam.12389. 25580768

[bib5] Adamiak JW, Bergen C, Ajmal L et al. Functional interplay between RND efflux pumps and GacS in *Pseudomonas aeruginosa*. Appl Environ Microbiol. 2025;91:e0122325. 10.1128/aem.01223-25. 40824111 PMC12442375

[bib6] Aggarwal C, Jimenez JC, Nanavati D et al. Multiple length peptide-pheromone variants produced by *Streptococcus pyogenes* directly bind Rgg proteins to confer transcriptional regulation. J Biol Chem. 2014;289:22427–36. 10.1074/jbc.M114.583989. 24958729 PMC4139249

[bib7] Anderson AJ, Kang BR, Kim YC. The Gac/Rsm signaling pathway of a biocontrol bacterium, *Pseudomonas chlororaphis* O6. Res Plant Dis. 2017;23:212–27.

[bib8] Anju VT, Busi S, Imchen M et al. Polymicrobial infections and biofilms: clinical significance and eradication strategies. Antibiotics. 2022;11:1731. 10.3390/antibiotics11121731. 36551388 PMC9774821

[bib9] Arcan SKC, Yatip P, Munyoo B et al. Attenuating *Vibrio harveyi* virulence through quorum sensing interference using piperine: an *in vitro* and *in silico* approach. J Fish Dis. 2025;48:14094. 10.1111/jfd.14094. 39907168

[bib10] Armbruster CE, Hong W, Pang B et al. Indirect pathogenicity of *Haemophilus influenzae* and *Moraxella catarrhalis* in polymicrobial otitis media occurs via interspecies quorum signaling. mBio. 2010;1:e00102–00110. 10.1128/mBio.00102-10. 20802829 PMC2925075

[bib11] Bahari S, Zeighami H, Mirshahabi H et al. Inhibition of *Pseudomonas aeruginosa* quorum sensing by subinhibitory concentrations of curcumin with gentamicin and azithromycin. J Glob Antimicrobial Resist. 2017;10:21–28. 10.1016/j.jgar.2017.03.006. 28591665

[bib12] Baldelli Valerio. D’Angelo Francesca. Pavoncello Viola et al. Identification of FDA-approved antivirulence drugs targeting thePseudomonas aeruginosaquorum sensing effector protein PqsE. Virulence. 2020;11:652–68. 10.1080/21505594.2020.1770508 32423284 PMC7549961

[bib13] Bandyopadhaya A, Tzika AA, Rahme LG. *Pseudomonas aeruginosa* quorum sensing molecule alters skeletal muscle protein homeostasis by perturbing the antioxidant defense system. mBio. 2019;10:e02211–02219. 31575771 10.1128/mBio.02211-19 PMC6775459

[bib14] Banerji R, Karkee A, Kanojiya P et al. Bacterial communication in the regulation of stress response in *Listeria monocytogenes*. LWT. 2022;154:112703. 10.1016/j.lwt.2021.112703.

[bib15] Barber JN, Nicholson LC, Woods LC et al. Species interactions constrain adaptation and preserve ecological stability in an experimental microbial community. ISME J. 2022;16:1442–52. 10.1038/s41396-022-01191-1. 35066567 PMC9039033

[bib16] Beenker WA, Hoeksma J, Bannier-Hélaouët M et al. Paecilomycone inhibits quorum sensing in Gram-negative bacteria. Microbiol Spectr. 2023;11:e05097–22. 10.1128/spectrum.05097-22. 36920212 PMC10100902

[bib17] Bejder BS, Monda F, Gless BH et al. A short-lived peptide signal regulates cell-to-cell communication in *Listeria monocytogenes*. Commun Biol. 2024;7:942. 10.1038/s42003-024-06623-6. 39097633 PMC11297923

[bib18] Bez C, Geller AM, Levy A et al. Cell-cell signaling proteobacterial LuxR solos: a treasure trove of subgroups having different origins, ligands, and ecological roles. mSystems. 2023;8:e01039–01022. 10.1128/msystems.01039-22. 36802056 PMC10134790

[bib19] Bisht K, Baishya J, Wakeman CA. *Pseudomonas aeruginosa* polymicrobial interactions during lung infection. Curr Opin Microbiol. 2020;53:1–8. 10.1016/j.mib.2020.01.014. 32062024 PMC7244363

[bib20] Bofinger MR, de Sousa LS, Fontes JEN et al. Diketopiperazines as cross-communication quorum-sensing signals between *Cronobacter sakazakii* and *Bacillus cereus*. ACS Omega. 2017;2:1003–8. 10.1021/acsomega.6b00513. 30023625 PMC6044783

[bib21] Boon C, Deng Y, Wang L et al. A novel DSF-like signal from *Burkholderia cenocepacia* interferes with *C and ida albicans* morphological transition. Int Soc Microbial Ecol J. 2008;2:27–36. 10.1038/ismej.2007.76 18049456

[bib22] Brackman G, Cos P, Maes L et al. Quorum sensing inhibitors increase the susceptibility of bacterial biofilms to antibiotics *in vitro* and *in vivo*. Antimicrob Agents Chemother. 2011;55:2655–61. 10.1128/AAC.00045-11. 21422204 PMC3101409

[bib23] Brameyer S, Heermann R. Quorum sensing and LuxR Solos in *Photorhabdus*. Curr Top Microbiol Immunol. 2017;402:103–19. 27848037 10.1007/82_2016_28

[bib24] Brameyer S, Kresovic D, Bode HB et al. LuxR solos in *Photorhabdus* species. Front Cell Infect Microbiol. 2014;4:166. 10.3389/fcimb.2014.00166. 25478328 PMC4235431

[bib25] Brameyer S, Kresovic D, Bode HB et al. Dialkylresorcinols as bacterial signaling molecules. Proc Natl Acad Sci USA. 2015;112:572–7. 10.1073/pnas.1417685112. 25550519 PMC4299209

[bib26] Bratu S, Gupta J, Quale J. Expression of the las and rhl quorum-sensing systems in clinical isolates of *Pseudomonas aeruginosa* does not correlate with efflux pump expression or antimicrobial resistance. J Antimicrob Chemother. 2006;58:1250–3. 10.1093/jac/dkl407. 17030516

[bib27] Buchholtz C, Nielsen KF, Milton DL et al. Profiling of acylated homoserine lactones of *Vibrio anguillarum* in vitro and in vivo: influence of growth conditions and serotype. Syst Appl Microbiol. 2006;29:433–45. 10.1016/j.syapm.2005.12.007. 16413159

[bib28] Cao H, Xia T, Li Y et al. Uncoupled quorum sensing modulates the interplay of virulence and resistance in a multidrug-resistant clinical *Pseudomonas aeruginosa* isolate belonging to the MLST550 clonal complex. Antimicrob Agents Chemother. 2019;63:e01944–18. 10.1128/AAC.01944-18. 30670423 PMC6437519

[bib29] Cao Y, He S, Zhou Z et al. Orally administered thermostable *N*-acyl homoserine lactonase from *Bacillus* sp. strain AI96 attenuates *Aeromonas hydrophila* infection in zebrafish. Appl Environ Microbiol. 2012;78:1899–908. 10.1128/AEM.06139-11. 22247159 PMC3298178

[bib30] Chambonnier G, Roux L, Redelberger D et al. The hybrid histidine kinase LadS forms a multicomponent signal transduction system with the GacS/GacA two-component system in *Pseudomonas aeruginosa*. PLoS Genet. 2016;12:e1006032. 10.1371/journal.pgen.1006032. 27176226 PMC4866733

[bib31] Chen CN, Chen CJ, Liao CT et al. A probable aculeacin A acylase from the *Ralstonia solanacearum* GMI1000 is N-acyl-homoserine lactone acylase with quorum-quenching activity. BMC Microbiol. 2009;9:89. 10.1186/1471-2180-9-89. 19426552 PMC2686713

[bib32] Chen Y, B and yopadhyay A, Kozlowicz BK et al. Mechanisms of peptide sex pheromone regulation of conjugation in *Enterococcus faecalis*. Microbiologyopen. 2017;6:e492. 10.1002/mbo3.492. PMC5552905 28523739

[bib33] Cheng WJ, Zhou JW, Zhang PP et al. Quorum sensing inhibition and tobramycin acceleration in *Chromobacterium violaceum* by two natural cinnamic acid derivatives. Appl Microbiol Biotechnol. 2020;104:5025–37. 10.1007/s00253-020-10593-0. 32248442

[bib34] Cheung GY, Wang R, Khan BA et al. Role of the accessory gene regulator agr in community-associated methicillin-resistant *Staphylococcus aureus* pathogenesis. Infect Immun. 2011;79:1927–35. 10.1128/IAI.00046-11. 21402769 PMC3088142

[bib35] Chowdhury S, Castro S, Coker C et al. Programmable bacteria induce durable tumor regression and systemic antitumor immunity. Nat Med. 2019;25:1057–63. 10.1038/s41591-019-0498-z. 31270504 PMC6688650

[bib36] Chua KO, S-T WS, Ee R et al. *In silico* analysis reveals distribution of quorum sensing genes and consistent presence of LuxR solos in the *Pandoraea* species. Front Microbiol. 2019;10:1758. 10.3389/fmicb.2019.01758. 31447806 PMC6691176

[bib37] Condori S, Atkinson S, Leys N et al. Construction and phenotypic characterization of M68, an RruI quorum sensing knockout mutant of the photosynthetic alphaproteobacterium *Rhodospirillum rubrum*. Res Microbiol. 2016;167:380–92. 10.1016/j.resmic.2016.02.006. 26993754

[bib38] Cui BB, Peng GJ, Wang MF et al. Carnosol attenuates *Acinetobacter baumannii* virulence by interfering with indole-mediated quorum sensing. Virulence. 2025;16:2530169. 10.1080/21505594.2025.2530169. 40660701 PMC12269709

[bib39] Cui S, Kim E. Quorum sensing and antibiotic resistance in polymicrobial infections. Commun Integr Biol. 2024;17:2415598. 10.1080/19420889.2024.2415598. 39430726 PMC11487952

[bib40] Dago AE, Schug A, Procaccini A et al. Structural basis of histidine kinase autophosphorylation deduced by integrating genomics, molecular dynamics, and mutagenesis. Proc Natl Acad Sci USA. 2012;109:1733–42. 10.1073/pnas.1201301109. PMC3387055 22670053

[bib41] Dal A, Hekimoğlu R, Sümbül B et al. Quorum-sensing inhibition by furanone compounds and therapeutic effects on *Pseudomonas aeruginosa* keratitis rabbit model. BMC Ophthalmol. 2025;25:231. 10.1186/s12886-025-04061-4. 40264027 PMC12013088

[bib42] D’Angelo F, Baldelli V, Halliday N et al. Identification of FDA-approved drugs as antivirulence agents targeting the pqs quorum-sensing system of *Pseudomonas aeruginosa*. Antimicrob Agents Chemother. 2018;62:e01296–01218. 10.1128/AAC.01296-18. 30201815 PMC6201120

[bib43] Darby EM, Trampari E, Siasat P et al. Molecular mechanisms of antibiotic resistance revisited. Nat Rev Microbiol. 2023;21:280–95. 10.1038/s41579-022-00820-y. 36411397

[bib44] Deep A, Chaudhary U, Gupta V. Quorum sensing and bacterial pathogenicity: from molecules to disease. J Lab Phys. 2011;3:4–11. 10.4103/0974-2727.78553 PMC3118056 21701655

[bib45] Deng Y, Wu J, Tao F et al. Listening to a new language: dSF-based quorum sensing in Gram-negative bacteria. Chem Rev. 2011;111:160–73. 10.1021/cr100354f. 21166386

[bib46] Desouky SE, Shojima A, Singh RP et al. Cyclodepsipeptides produced by actinomycetes inhibit cyclic-peptide-mediated quorum sensing in Gram-positive bacteria. FEMS Microbiol Lett. 2015;362:fnv109. 10.1093/femsle/fnv109. 26149266

[bib47] Dimitrova PD, Damyanova T, Paunova-Krasteva T. *Chromobacterium violaceum*: a model for evaluating the anti-quorum sensing activities of plant substances. Sci Pharm. 2023;91:33. 10.3390/scipharm91030033.

[bib48] Dong K, Pan H, Yang D et al. Induction, detection, formation, and resuscitation of viable but non-culturable state microorganisms. Comp Rev Food Sci Food Safe. 2020;19:149–83. 10.1111/1541-4337.12513. 33319518

[bib49] Dong X, Tu C, Liu Y et al. Identification of the core c-di-GMP turnover proteins responsible for root colonization of *Bacillus velezensis*. iScience. 2022;25:105294. 10.1016/j.isci.2022.105294. 36300004 PMC9589206

[bib50] Dow JM, Naughton LM, Hollmann B et al. The diffusible signal factor family of bacterial cell-cell Signals. Isr J Chem. 2016;56:321–9. 10.1002/ijch.201500075.

[bib51] Fan Q, Wang H, Mao C et al. Structure and signal regulation mechanism of interspecies and Interkingdom quorum sensing system receptors. J Agric Food Chem. 2022;70:429–45. 10.1021/acs.jafc.1c04751. 34989570

[bib52] Fang Z, Sun D, Li C et al. Regulatory effects of *Shewanella putrefaciens* isolated from shrimp *Penaeus orientalis* on the virulence factors of *Vibrio parahaemolyticus* and evaluation of the role of quorum sensing in virulence factors regulation. FEMS Microbiol Ecol. 2018;94:fly097. 10.1093/femsec/fiy097. 29800146

[bib53] Freeman ZN, Dorus S, Waterfield NR. The KdpD/KdpE two-component system: integrating K⁺ homeostasis and virulence. PLoS Pathog. 2013;9:e1003201. 10.1371/journal.ppat.1003201. 23555240 PMC3610689

[bib54] Fuqua WC, Winans SC, Greenberg EP. Quorum sensing in bacteria: the LuxR-LuxI family of cell density-responsive transcriptional regulators. J Bacteriol. 1994;176:269–75. 10.1128/jb.176.2.269-275.1994. 8288518 PMC205046

[bib55] Furiga A, Lajoie B, El Hage S et al. Impairment of *Pseudomonas aeruginosa* biofilm resistance to antibiotics by combining the drugs with a new quorum-sensing inhibitor. Antimicrob Agents Chemother. 2015;60:1676–86. 10.1128/AAC.02533-15. 26711774 PMC4775964

[bib56] Gao XY, Fu CA, Hao L et al. The substrate-dependent regulatory effects of the AfeI/R system in *Acidithiobacillus ferrooxidans* reveals the novel regulation strategy of quorum sensing in acidophiles. Environ Microbiol. 2021;23:757–73. 10.1111/1462-2920.15163. 32656931 PMC7984328

[bib57] Garmyn D, Gal L, Bri and et R et al. Evidence of autoinduction heterogeneity via expression of the Agr system of *Listeria monocytogenes* at the single-cell level. Appl Environ Microbiol. 2011;77:6286–9. 10.1128/AEM.02891-10. 21724873 PMC3165395

[bib58] Gless BH, Peng P, Pedersen KD et al. Structure-activity relationship study based on autoinducing peptide (AIP) from dog pathogen *S. schleiferi*. Org Lett. 2017;19:5276–9. 10.1021/acs.orglett.7b02550. 28952740

[bib59] Guo Q, Zhang Y, Fang X et al. Two-component system *virS*/*virR* regulated biofilm formation of *Listeria monocytogenes* 10403S. Food Biosci. 2023;55:102973. 10.1016/j.fbio.2023.102973.

[bib60] Guo Y, Gao M, Wang L et al. An engineered probiotic consortium based on quorum-sensing for colorectal cancer immunotherapy. Adv Sci. 2025;12:e12744. 10.1002/advs.202512744. PMC12697889 40999885

[bib61] Haque M, Islam S, Sheikh MA et al. Quorum sensing: a new prospect for the management of antimicrobial-resistant infectious diseases. Expert Rev Anti Infect Ther. 2021;19:571–86. 10.1080/14787210.2021.1843427. 33131352

[bib62] Helcman M, Smejkal K, Culenová M et al. Natural phenolics disrupt microbial communication by inhibiting quorum sensing. Microorganisms. 2025;13:287. 10.3390/microorganisms13020287. 40005654 PMC11857621

[bib63] Henke JM, Bassler BL. Quorum sensing regulates type III secretion in *Vibrio harveyi* and *Vibrio parahaemolyticus*. J Bacteriol. 2004;186:3794–805. 10.1128/JB.186.12.3794-3805.2004. 15175293 PMC419960

[bib64] Hermanas TM, Subramanian S, Dann CE III et al. Spore-associated proteins involved in c-di-GMP synthesis and degradation of *Bacillus anthracis*. J Bacteriol. 2021;203:e00135–21. 10.1128/JB.00135-21. 34096779 PMC8351633

[bib65] Higgins DA, Pomianek ME, Kraml CM et al. The major *Vibrio cholerae* autoinducer and its role in virulence factor production. Nature. 2007;450:883–6. 10.1038/nature06284. 18004304

[bib66] Higgins S, Heeb S, Rampioni G et al. Differential regulation of the phenazine biosynthetic operons by quorum sensing in *Pseudomonas aeruginosa* PAO1-N. Front Cell Infect Microbiol. 2018;8:252. 10.3389/fcimb.2018.00252. 30083519 PMC6064868

[bib67] Hoover SE, Amilcar JP, Tsu H-CT et al. A new quorum-sensing system (TprA/PhrA) for *Streptococcus pneumoniae* D39 that regulates a lantibiotic biosynthesis gene cluster. Mol Microbiol. 2015;97:229–43. 10.1111/mmi.13029. 25869931 PMC4676566

[bib68] Hu C, He G, Yang Y et al. Nanomaterials regulate bacterial quorum sensing: applications, mechanisms, and optimization strategies. Adv Sci. 2024;11:e2306070. 10.1002/advs.202306070. PMC11022734 38350718

[bib69] Huang J, Wang R, Zhang Q et al. Positive regulation of the PhcB neighbouring regulator PrhX on expression of the type III secretion system and pathogenesis in *Ralstonia solanacearum*. Mol Plant Pathol. 2024;25:e13398. 10.1111/mpp.13398. 37877898 PMC10788593

[bib70] Huang X, Duddy OP, Silpe JE et al. Mechanism underlying autoinducer recognition in the *Vibrio cholerae* DPO-VqmA quorum-sensing pathway. J Biol Chem. 2020;295:2916–31. 10.1074/jbc.RA119.012104. 31964715 PMC7062168

[bib71] Huang Y, Chen Y, Zhang LH. The roles of microbial cell-cell chemical communication systems in the modulation of antimicrobial resistance. Antibiotics. 2020;9:779. 10.3390/antibiotics9110779. 33171916 PMC7694446

[bib72] Huedo P, Kumar V, Horgan C et al. Sulfonamide-based diffusible signal factor analogs interfere with quorum sensing in *Stenotrophomonas maltophilia* and *Burkholderia cepacia*. Future Med Chem. 2019;11:1565–82. 10.4155/fmc-2019-0015. 31469336

[bib73] Huedo P, Yero D, Martinez-Servat S et al. Two different rpf clusters distributed among a population of *Stenotrophomonas maltophilia* clinical strains display differential diffusible signal factor production and virulence regulation. J Bacteriol. 2014;196:2431–42. 10.1128/JB.01540-14. 24769700 PMC4054175

[bib74] Huillet E, Bridoux L, Barboza I et al. The signaling peptide PapR is required for the activity of the quorum-sensor PlcRa in *Bacillus thuringiensis*. Microbiology. 2020;166:398–410. 10.1099/mic.0.000883. 32067627

[bib75] Inagaki R, Koshiba A, Nasuno E et al. Eliminating extracellular autoinducing peptide signals inhibits the *Staphylococcus aureus* quorum sensing agr system. Biochem Biophys Res Commun. 2024;711:149912. 10.1016/j.bbrc.2024.149912. 38615572

[bib76] Ivanova K, Fern and es MM, Mendoza E et al. Enzyme multilayer coatings inhibit *Pseudomonas aeruginosa* biofilm formation on urinary catheters. Appl Microbiol Biotechnol. 2015;99:4373–85. 10.1007/s00253-015-6378-7. 25582561

[bib77] Jeon B, Itoh K. Production of shiga toxin by a luxS mutant of *Escherichia coli* O157:H7 in vivo and in vitro. Microbiol Immunol. 2007;51:391–6. 10.1111/j.1348-0421.2007.tb03926.x. 17446678

[bib78] Ji HR, Zhao L, Lv KW et al. Citrinin is a potential quorum sensing inhibitor against *Pseudomonas aeruginosa*. Mar Drugs. 2023;21:296. 10.3390/md21050296. 37233490 PMC10221562

[bib79] Jia T, Bi X, Li M et al. Hfq-binding small RNA PqsS regulates *Pseudomonas aeruginosa* pqs quorum sensing system and virulence. npj Biofilms Microbiomes. 2024;10:82. 10.1038/s41522-024-00550-4. 39261499 PMC11391009

[bib80] Jiang. J, Liu. H, Xiao. H et al. Cloning and identification of *hdtS* gene from *Oxalobacter formigenes* encoding an acylhomoserine lactone synthase of quorum-sensing system. J Northeast Agric Univ. 2014;45:49–56.

[bib81] Jin L, Zhang X, Shi H et al. Identification of a novel *N*-acyl homoserine lactone synthase, AhyI, in *Aeromonas hydrophila* and structural basis for its substrate specificity. J Agric Food Chem. 2020;68:2516–27. 10.1021/acs.jafc.9b07833. 32050067

[bib82] Jung H, Kim Y, Lee Y et al. Mutation of the cyclic di-GMP phosphodiesterase gene in *Burkholderia lata* SK875 attenuates virulence and enhances biofilm formation. J Microbiol. 2017;55:800–8. 10.1007/s12275-017-7374-7. 28956352

[bib83] Juszczuk-Kubiak E . Molecular aspects of the functioning of pathogenic bacteria biofilm based on quorum sensing (QS) signal-response system and innovative non-antibiotic strategies for their elimination. Int J Mol Sci. 2024;25:2655. 10.3390/ijms25052655. 38473900 PMC10931677

[bib84] Kai K, Ohnishi H, Shimatani M et al. Methyl 3-hydroxymyristate, a diffusible signal mediating *phc* quorum sensing in *Ralstonia solanacearum*. ChemBioChem. 2015;16:2309–18. 10.1002/cbic.201500456. 26360813

[bib85] Kai K . The *phc* quorum-sensing system in *Ralstonia solanacearum* species complex. Annu Rev Microbiol. 2023;77:213–31. 10.1146/annurev-micro-032521-030537. 37100406

[bib86] Kalia VC, Wood TK, Kumar P. Evolution of resistance to quorum-sensing inhibitors. Microb Ecol. 2014;68:13–23. 10.1007/s00248-013-0316-y. 24194099 PMC4012018

[bib87] Kareb O, Aïder M. Quorum sensing circuits in the communicating mechanisms of bacteria and its implication in the biosynthesis of bacteriocins by lactic acid Bacteria: a review. Probiotics Antimicro Prot. 2020;12:5–17. 10.1007/s12602-019-09555-4. 31104210

[bib88] Kaushik V, Sharma S, Tiwari M et al. Antipersister strategies against stress induced bacterial persistence. Microb Pathog. 2022;164:105423. 10.1016/j.micpath.2022.105423. 35092834

[bib89] Ke X, Miller L, Ng W-L et al. CqsA-CqsS quorum-sensing signal-receptor specificity in *Photobacterium angustum*. Mol Microbiol. 2014;91:821–33. 10.1111/mmi.12502. 24372841 PMC3959898

[bib90] Khayat MT, Abbas HA, Ibrahim TS et al. Anti-quorum sensing activities of gliptins against *Pseudomonas aeruginosa* and *Staphylococcus aureus*. Biomedicines. 2022;10:1169. 10.3390/biomedicines10051169. 35625906 PMC9138634

[bib91] Kim CS, Gatsios A, Cuesta S et al. Characterization of Autoinducer-3 structure and biosynthesis in *E. coli*. ACS Cent Sci. 2020;6:197–206. 10.1021/acscentsci.9b01076. 32123737 PMC7047286

[bib92] Kim JA, Jang BR, Kim YR et al. *Vibrio vulnificus* induces the death of a major bacterial species in the mouse gut via cyclo-Phe-Pro. Microbiome. 2021;9:161. 10.1186/s40168-021-01095-w. 34284824 PMC8293591

[bib93] Kong W, Chen L, Zhao J et al. Hybrid sensor kinase PA1611 in *Pseudomonas aeruginosa* regulates transitions between acute and chronic infection through direct interaction with RetS. Mol Microbiol. 2013;88:784–97. 10.1111/mmi.12223. 23560772

[bib94] Korgaonkar A, Trivedi U, Rumbaugh KP et al. Community surveillance enhances *Pseudomonas aeruginosa* virulence during polymicrobial infection. Proc Natl Acad Sci USA. 2013;110:1059–64. 10.1073/pnas.1214550110. 23277552 PMC3549110

[bib95] Koul S, Prakash J, Mishra A et al. Potential emergence of multi-quorum sensing inhibitor resistant (MQSIR) bacteria. Ind J Microbiol. 2016;56:1–18. 10.1007/s12088-015-0558-0. PMC4729740 26843692

[bib96] Kovacevic J, Ziegler J, Wałecka-Zacharska E et al. Tolerance of *Listeria monocytogenes* to quaternary ammonium sanitizers is mediated by a novel efflux pump encoded by *emrE*. Appl Environ Microbiol. 2016;82:939–53. 10.1128/AEM.03741-15. 26590290 PMC4725271

[bib97] Kresovic D, Schempp F, Cheikh-Ali Z et al. A novel and widespread class of ketosynthase is responsible for the head-to-head condensation of two acyl moieties in bacterial pyrone biosynthesis. Beilstein J Org Chem. 2015;11:1412–7. 10.3762/bjoc.11.152. 26425196 PMC4578411

[bib98] Kumar JS, Umesha S, Prasad KS et al. Detection of quorum sensing molecules and biofilm formation in *Ralstonia solanacearum*. Curr Microbiol. 2016;72:297–305. 26620535 10.1007/s00284-015-0953-0

[bib99] Kumar L, Patel SKS, Kharga K et al. Molecular mechanisms and applications of N-acyl homoserine lactone-mediated quorum sensing in bacteria. Molecules. 2022;27:7584. 10.3390/molecules27217584. 36364411 PMC9654057

[bib100] Lallement C, Goldring WPD, Jelsbak L. Global transcriptomic response of the AI-3 isomers 3,5-DPO and 3,6-DPO in *Salmonella Typhimurium*. Arch Microbiol. 2023;205:117. 10.1007/s00203-023-03450-x. 36929450

[bib101] Laue BE, Jiang Y, Chhabra SR et al. The biocontrol strain *Pseudomonas fluorescens* F113 produces the rhizobium small bacteriocin,N-(3-hydroxy-7-cis-tetradecenoyl)homoserine lactone, via HdtS, a putative novel *N*-acylhomoserine lactone synthase. Microbiology. 2000;146:2469–80. 10.1099/00221287-146-10-2469. 11021923

[bib102] Lee MH, Khan R, Tao W et al. Soil metagenome-derived 3-hydroxypalmitic acid methyl ester hydrolases suppress extracellular polysaccharide production in *Ralstonia solanacearum*. J Biotechnol. 2018;270:30–38. 10.1016/j.jbiotec.2018.01.023. 29407418

[bib103] Li D-d, Y W, Kim EL et al. Neuroprotective effect of cyclo-(L-Pro-L-Phe) isolated from the jellyfish-derived fungus *Aspergillus flavus*. Mar Drugs. 2021;19:417. 10.3390/md19080417. 34436256 PMC8401322

[bib104] Li P, Yin W, Yan J et al. Modulation of inter-kingdom communication by PhcBSR quorum sensing system in *Ralstonia solanacearum* phylotype I strain GMI1000. Front Microbiol. 2017;8:1172. 10.3389/fmicb.2017.01172. 28690607 PMC5481312

[bib105] Li X, Liu Y, Wang Y et al. Resistance risk induced by quorum sensing inhibitors and their combined use with antibiotics: mechanism and its relationship with toxicity. Chemosphere. 2021;265:129153. 10.1016/j.chemosphere.2020.129153. 33302207

[bib106] Li X, Yin W, Lin JD et al. Regulation of the physiology and virulence of *Ralstonia solanacearum* by the second messenger 2′,3′-cyclic guanosine monophosphate. Nat Commun. 2023;14:7654. 10.1038/s41467-023-43461-2. 37996405 PMC10667535

[bib107] Li Y, Feng T, Wang Y. The role of bacterial signaling networks in antibiotics response and resistance regulation. Mar Life Sci Technol. 2022;4:163–78. 10.1007/s42995-022-00126-1. 37073223 PMC10077285

[bib108] Liang H, Feng YM, Zeng D et al. DSF-inspired discovery of novel zingerone-based quorum-sensing inhibitors: an attractive tactic of fighting *Xanthomonas* bacterial infections. Pest Manage Sci. 2025;81:4348–64. 10.1002/ps.8793. 40135465

[bib109] Ling J, Zhu R, Laborda P et al. LbDSF, the *Lysobacter brunescens* quorum-sensing system diffusible signaling factor, regulates anti- *Xanthomonas* XSAC biosynthesis, colony morphology, and surface motility. Front Microbiol. 2019;10:1230. 10.3389/fmicb.2019.01230. 31275253 PMC6591275

[bib110] Liu L, Tan X, Jia A. Relationship between bacterial quorum sensing and biofilm formation—a review. Acta Microbiol Sin. 2012;52:271–8. 22712396

[bib111] Liu L, Yan Y, Feng L et al. Quorum sensing asaI mutants affect spoilage phenotypes, motility, and biofilm formation in a marine fish isolate of *Aeromonas salmonicida*. Food Microbiol. 2018;76:40–51. 10.1016/j.fm.2018.04.009. 30166167

[bib112] Liu L, Zeng X, Zheng J et al. AHL-mediated quorum sensing to regulate bacterial substance and energy metabolism: a review. Microbiol Res. 2022;262:127102. 10.1016/j.micres.2022.127102. 35792523

[bib113] Liu X, Wang W, Li J et al. A widespread response of Gram-negative bacterial acyl-homoserine lactone receptors to Gram-positive *Streptomyces* γ-butyrolactone signaling molecules. Sci Chin Life Sci. 2021;64:1575–89. 10.1007/s11427-021-1956-8. 34319534

[bib114] Luján AM, Paterson S, Hesse E et al. Polymicrobial infections can select against *Pseudomonas aeruginosa* mutators because of quorum-sensing trade-offs. Nat Ecol Evol. 2022;6:979–88. 10.1038/s41559-022-01768-1. 35618819

[bib115] Luo J, Dong B, Wang K et al. Baicalin inhibits biofilm formation, attenuates the quorum sensing-controlled virulence and enhances *Pseudomonas aeruginosa* clearance in a mouse peritoneal implant infection model. PLoS One. 2017;12:e0176883. 10.1371/journal.pone.0176883. 28453568 PMC5409170

[bib116] Luo Q, Shang J, Feng X et al. PrfA led to reduced biofilm formation and contributed to altered gene expression patterns in biofilm-forming *Listeria monocytogenes*. Curr Microbiol. 2013;67:372–8. 10.1007/s00284-013-0377-7. 23652633

[bib117] Mai T, Tintillier F, Lucasson A et al. Quorum sensing inhibitors from *Leucetta chagosensis* Dendy, 1863. Lett Appl Microbiol. 2015;61:311–7. 10.1111/lam.12461. 26138555

[bib118] Maiga A, Ampomah-Wireko M, Li H et al. Multidrug-resistant bacteria quorum-sensing inhibitors: a particular focus on *Pseudomonas aeruginosa*. Eur J Med Chem. 2025;281:117008. 10.1016/j.ejmech.2024.117008. 39500066

[bib119] Mandabi A, Ganin H, Meijler MM. Synergistic activation of quorum sensing in *Vibrio harveyi*. Bioorg Med Chem Lett. 2015;25:3966–9. 26248803 10.1016/j.bmcl.2015.07.028

[bib120] Mangwani N, Dash HR, Chauhan A et al. Bacterial quorum sensing: functional features and potential applications in biotechnology. J Mol Microbiol Biotechnol. 2012;22:215–27. 22964521 10.1159/000341847

[bib121] Mayville P, Ji G, Beavis R et al. Structure-activity analysis of synthetic autoinducing thiolactone peptides from *Staphylococcus aureus* responsible for virulence. Proc Natl Acad Sci USA. 1999;96:1218–23. 10.1073/pnas.96.4.1218. 9990004 PMC15443

[bib122] McBrayer DN, Cameron CD, Gantman BK et al. Rational design of potent activators and inhibitors of the *Enterococcus faecalis* Fsr quorum sensing circuit. ACS Chem Biol. 2018;13:2673–81. 10.1021/acschembio.8b00610. 30141904 PMC6150820

[bib123] Meijer WJ, Boer DR, Ares S et al. Multiple layered control of the conjugation process of the *Bacillus subtilis* plasmid pLS20. Front Mol Biosci. 2021;8:648468. 10.3389/fmolb.2021.648468. 33816561 PMC8014075

[bib124] Meireles D, Pombinho R, Cabanes D. Signals behind *Listeria monocytogenes* virulence mechanisms. Gut Microbes. 2024;16:2369564. 10.1080/19490976.2024.2369564. 38979800 PMC11236296

[bib125] Mi JQ, Yu ZY, Yu H et al. Quorum sensing systems in foodborne *Salmonella* spp. and corresponding control strategies using quorum sensing inhibitors for food storage. Trends Food Sci Technol. 2024;144:104320. 10.1016/j.tifs.2023.104320.

[bib126] Milton DL, Chalker VJ, Kirke D et al. The LuxM homologue VanM from *Vibrio anguillarum* directs the synthesis of *N*-(3-hydroxyhexanoyl)homoserine lactone and *N*-hexanoylhomoserine lactone. J Bacteriol. 2001;183:3537–47. 10.1128/JB.183.12.3537-3547.2001. 11371516 PMC95229

[bib127] Mina G, Chbib C. Recent progresses on synthesized LuxS inhibitors: a mini-review. Bioorg Med Chem. 2019;27:36–42. 10.1016/j.bmc.2018.11.026. 30473360

[bib128] Mion S, Rémy B, Plener L et al. Quorum quenching lactonase strengthens bacteriophage and antibiotic arsenal against *Pseudomonas aeruginosa* clinical isolates. Front Microbiol. 2019;10:2049. 10.3389/fmicb.2019.02049. 31551983 PMC6734170

[bib129] Miranda V, Torcato IM, Xavier KB et al. Synthesis of d-desthiobiotin-AI-2 as a novel chemical probe for autoinducer-2 quorum sensing receptors. Bioorg Chem. 2019;92:103200. 31470199 10.1016/j.bioorg.2019.103200

[bib130] Mukherjee S, Bassler BL. Bacterial quorum sensing in complex and dynamically changing environments. Nat Rev Microbiol. 2019;17:371–82. 10.1038/s41579-019-0186-5. 30944413 PMC6615036

[bib131] Murray EJ, Dubern JF, Chan WC et al. A *Pseudomonas aeruginosa* PQS quorum-sensing system inhibitor with anti-staphylococcal activity sensitizes polymicrobial biofilms to tobramycin. Cell Chem Biol. 2022;29:1187–99.e6. 10.1016/j.chembiol.2022.02.007. 35259345 PMC9605878

[bib132] Nagi M, Chapple ILC, Sharma P et al. Quorum sensing in oral biofilms: influence on host cells. Microorganisms. 2023;11:1688. 10.3390/microorganisms11071688. 37512861 PMC10386421

[bib133] Neiditch MB, Capodagli GC, Prehna G et al. Genetic and structural analyses of RRNPP intercellular peptide signaling of Gram-positive bacteria. Annu Rev Genet. 2017;51:311–33. 10.1146/annurev-genet-120116-023507. 28876981 PMC6588834

[bib134] Nordgaard M, Mortensen RMR, Kirk NK et al. Deletion of Rap-Phr systems in *Bacillus subtilis* influences *in vitro* biofilm formation and plant root colonization. Microbiologyopen. 2021;10:e1212. 10.1002/mbo3.1212. 34180604 PMC8236291

[bib135] Nyffeler KE, Santa EE, Blackwell HEJA. Small molecules with either receptor-selective or Pan-receptor activity in the three LuxR-type receptors that regulate quorum sensing in *Pseudomonas aeruginosa*. ACS Chem Biol. 2022;17:2979–85. 10.1021/acschembio.2c00675. 36239990 PMC9675725

[bib136] Orazi G, O’Toole GA. “It Takes a Village”: mechanisms underlying antimicrobial recalcitrance of polymicrobial biofilms. J Bacteriol. 2019;202:e00530–19. 10.1128/JB.00530-19. 31548277 PMC6932244

[bib137] Ouyang J, Sun F, Feng W et al. Quercetin is an effective inhibitor of quorum sensing, biofilm formation and virulence factors in *Pseudomonas aeruginosa*. J Appl Microbiol. 2016;120:966–74. 10.1111/jam.13073. 26808465

[bib138] Packiavathy IA, Priya S, P and ian SK et al. Inhibition of biofilm development of uropathogens by curcumin—an anti-quorum sensing agent from *Curcuma longa*. Food Chem. 2014;148:453–60. 10.1016/j.foodchem.2012.08.002. 24262582

[bib139] Papenfort K, Silpe JE, Schramma KR et al. A *Vibrio cholerae* autoinducer-receptor pair that controls biofilm formation. Nat Chem Biol. 2017;13:551–7. 10.1038/nchembio.2336. 28319101 PMC5391282

[bib140] Patel K, Panchal R, Sakariya B et al. Combatting antibiotic resistance by exploring the promise of Quorum Quenching in targeting bacterial virulence. The Microbe. 2025;6:100224. 10.1016/j.microb.2024.100224.

[bib141] Patel K, Rodriguez C, Stabb EV et al. Spatially propagating activation of quorum sensing in *Vibrio fischeri* and the transition to low population density. Phys Rev E. 2020;101:062421. 10.1103/PhysRevE.101.062421. 32688581

[bib142] Patel R, Soni M, Soyantar B et al. A clash of quorum sensing vs quorum sensing inhibitors: an overview and risk of resistance. Arch Microbiol. 2023;205:107. 10.1007/s00203-023-03442-x. 36881156

[bib143] Paul D, Gopal J, Kumar M et al. Nature to the natural rescue: silencing microbial chats. Chem Biol Interact. 2018;280:86–98. 10.1016/j.cbi.2017.12.018. 29247642

[bib144] Pulingam T, Parumasivam T, Gazzali AM et al. Antimicrobial resistance: prevalence, economic burden, mechanisms of resistance and strategies to overcome. Eur J Pharm Sci. 2022;170:106103. 10.1016/j.ejps.2021.106103. 34936936

[bib145] Qin XF, Vila-Sanjurjo C, Singh R et al. Screening of bacterial quorum sensing inhibitors in a *Vibrio fischeri* LuxR-Based synthetic fluorescent *E. coli* biosensor. Pharmaceuticals. 2020;13:263. 10.3390/ph13090263. 32971993 PMC7559085

[bib146] Rasmussen TB, Givskov M. Quorum-sensing inhibitors as anti-pathogenic drugs. Int J Med Microbiol. 2006;296:149–61. 10.1016/j.ijmm.2006.02.005. 16503194

[bib147] Rattanaphan P, Mittraparp-Arthorn P, Srinoun K et al. Indole signaling decreases biofilm formation and related virulence of *Listeria monocytogenes*. FEMS Microbiol Lett. 2020;367:fnaa116. 10.1093/femsle/fnaa116. 32658271

[bib148] Rebuffat S . Ribosomally synthesized peptides, foreground players in microbial interactions: recent developments and unanswered questions. Nat Prod Rep. 2022;39:273–310. 10.1039/D1NP00052G. 34755755

[bib149] Renye JA Jr, Somkuti GA. BlpC-regulated bacteriocin production in *Streptococcus thermophilus*. Biotechnol Lett. 2013;35:407–12. 10.1007/s10529-012-1095-0. 23183916

[bib150] Reuter K, Steinbach A, Helms V. Interfering with bacterial quorum sensing. Perspect Medicin Chem. 2016;8:1–15. 10.4137/PMC.S13209. 26819549 PMC4718088

[bib151] Rhodes KA, Schweizer HP. Antibiotic resistance in *Burkholderia* species. Drug Resist Updat. 2016;28:82–90. 10.1016/j.drup.2016.07.003. 27620956 PMC5022785

[bib152] Riedel CU, Monk IR, Casey PG et al. AgrD-dependent quorum sensing affects biofilm formation, invasion, virulence and global gene expression profiles in *Listeria monocytogenes*. Mol Microbiol. 2009;71:1177–89. 10.1111/j.1365-2958.2008.06589.x. 19154329

[bib153] Riga EK, Vöhringer M, Widyaya VT et al. Polymer-based surfaces designed to reduce biofilm formation: from antimicrobial polymers to strategies for long-term applications. Macromol Rapid Commun. 2017;38. 10.1002/marc.201700216. PMC7611510 28846821

[bib154] Rosic I, Nikolic I, Anteljevic M et al. Diversity and activity of AHL-lactonases in *Bacillus* spp. from various environments. FEMS Microbiol Lett. 2025;372:fnaf038. 10.1093/femsle/fnaf038. 40194945

[bib155] Rutherford ST, Bassler BL. Bacterial quorum sensing: its role in virulence and possibilities for its control. Cold Spring Harb Perspect Med. 2012;2:a012427. 10.1101/cshperspect.a012427. 23125205 PMC3543102

[bib156] Ryan GT, Wei Y, Winans SC. A LuxR-type repressor of *Burkholderia cenocepacia* inhibits transcription via antiactivation and is inactivated by its cognate acylhomoserine lactone. Mol Microbiol. 2013;87:94–111. 10.1111/mmi.12085. 23136852 PMC3535678

[bib157] Ryan RP, An S, Allan JH et al. The DSF family of cell–cell signals: an exp and ing class of bacterial virulence regulators. PLoS Pathog. 2015;11:1–14. 10.1371/journal.ppat.1004986. PMC4504480 26181439

[bib158] Sabir S, Das T, Kuppusamy R et al. Novel quinazolinone disulfide analogues as pqs quorum sensing inhibitors against *Pseudomonas aeruginosa*. Bioorg Chem. 2023;130:106226. 10.1016/j.bioorg.2022.106226. 36332317

[bib159] Sachdeva C, Satyamoorthy K, Murali TS. *Pseudomonas aeruginosa*: metabolic allies and adversaries in the world of polymicrobial infections. Crit Rev Microbiol. 2025;51:619–38. 10.1080/1040841X.2024.2397359. 39225080

[bib160] Sams T, Baker Y, Hodgkinson J et al. The *Pseudomonas* quinolone signal (PQS). Isr J Chem. 2016;56:282–94. 10.1002/ijch.201400128. PMC5477026 28660026

[bib161] Santajit S, Sookrung N, Indrawattana N. Quorum sensing in ESKAPE bugs: a target for combating antimicrobial resistance and bacterial virulence. Biology. 2022;11:1466. 10.3390/biology11101466. 36290370 PMC9598666

[bib162] Schutz C, Empting M. Targeting the *Pseudomonas* quinolone signal quorum sensing system for the discovery of novel anti-infective pathoblockers. Beilstein J Org Chem. 2018;14:2627–45. 10.3762/bjoc.14.241. 30410625 PMC6204780

[bib163] Sela R, Hammer BK, Halpern M. Quorum-sensing signaling by chironomid egg masses’ microbiota, affects haemagglutinin/protease (HAP) production by *Vibrio cholerae*. Mol Ecol. 2021;30:1736–46. 10.1111/mec.15662. 33001525

[bib164] Senuma W, Takemura C, Hayashi K et al. The putative sensor histidine kinase PhcK is required for the full expression of *phcA* encoding the global transcriptional regulator to drive the quorum-sensing circuit of *Ralstonia solanacearum* strain OE1-1. Mol Plant Pathol. 2020;21:1591–605. 10.1111/mpp.12998. 33025726 PMC7694676

[bib165] Shaaban M, Elgaml A, Habib EE. Biotechnological applications of quorum sensing inhibition as novel therapeutic strategies for multidrug resistant pathogens. Microb Pathog. 2019;127:138–43. 10.1016/j.micpath.2018.11.043. 30503958

[bib166] Shanker E, Federle MJ. Quorum sensing regulation of competence and bacteriocins in *Streptococcus pneumoniae* and mutans. Genes. 2017;8:15. 10.3390/genes8010015. 28067778 PMC5295010

[bib167] Sikdar R, Elias MH. Evidence for complex interplay between quorum sensing and antibiotic resistance in *Pseudomonas aeruginosa*. Microbiol Spectr. 2022;10:e0126922. 10.1128/spectrum.01269-22. 36314960 PMC9769976

[bib168] Singh R, Ray P. Quorum sensing-mediated regulation of *staphylococcal* virulence and antibiotic resistance. Future Microbiol. 2014;9:669–81. 10.2217/fmb.14.31. 24957093

[bib169] Sionov RV, Steinberg D. Targeting the holy triangle of quorum sensing, biofilm formation, and antibiotic resistance in pathogenic bacteria. Microorganisms. 2022;10:1239. 10.3390/microorganisms10061239. 35744757 PMC9228545

[bib170] Slamti L, Lemy C, Henry C et al. CodY regulates the activity of the virulence quorum sensor PlcR by controlling the import of the signaling peptide PapR in *Bacillus thuringiensis*. Front Microbiol. 2016;6:1501. 10.3389/fmicb.2015.01501. 26779156 PMC4701985

[bib171] Soheili V, Oghaz NK, Noughabi ZS et al. The novel effect of *cis*-2-decenoic acid on biofilm producing *Pseudomonas aeruginosa*. Microbiol Res. 2015;6:1–5.

[bib172] Song S, Yin W, Sun X et al. Anthranilic acid from *Ralstonia solanacearum* plays dual roles in intraspecies signalling and inter-kingdom communication. ISME J. 2020;14:2248–60. 10.1038/s41396-020-0682-7. 32457502 PMC7608240

[bib173] Song Z, Hou Y, Yang Q et al. Structures and biological activities of diketopiperazines from marine organisms: a review. Mar Drugs. 2021;19:403. 10.3390/md19080403. 34436242 PMC8398661

[bib174] Spacapan M, Danevcic T, Stefanic P et al. The ComX quorum sensing peptide of *Bacillus subtilis* affects biofilm formation negatively and sporulation positively. Microorganisms. 2020;8:1131. 10.3390/microorganisms8081131. 32727033 PMC7463575

[bib175] Su Y, M-y X, Cui Y et al. Bacterial quorum sensing orchestrates longitudinal interactions to shape microbiota assembly. Microbiome. 2023;11:241. 10.1186/s40168-023-01699-4. 37926838 PMC10626739

[bib176] Suresh S, Alva PP, Premanath R. Modulation of quorum sensing-associated virulence in bacteria: carbohydrate as a key factor. Arch Microbiol. 2021;203:1881–90. 10.1007/s00203-021-02235-4. 33641039

[bib177] Taj Z, Chattopadhyay I. *Staphylococcus aureus* virulence factors and biofilm components: synthesis, structure, function and inhibitors. In: Busi S, Prasad R (eds). ESKAPE Pathogens: Detection, Mechanisms and Treatment Strategies. Singapore: Springer Nature, 2004,227–70.

[bib178] Thoendel M, Kavanaugh JS, Flack CE et al. Peptide signaling in the *Staphylococci*. Chem Rev. 2011;111:117–51. 10.1021/cr100370n. 21174435 PMC3086461

[bib179] Tian X-Q, Wu Y, Cai Z et al. BDSF is a degradation-prone quorum-sensing signal detected by the histidine kinase RpfC of *Xanthomonas campestris* pv. campestris. Appl Environ Microbiol. 2022;88:e00031–00022. 10.1128/aem.00031-22. 35369702 PMC9040594

[bib180] Tonkin M, Khan S, Wani MY et al. Quorum sensing—a stratagem for conquering multi-drug resistant pathogens. CPD. 2021;27:2835–47. 10.2174/1381612826666201210105638. 33302856

[bib181] Topa SH, Palombo EA, Kingshott P et al. Activity of cinnamaldehyde on quorum sensing and biofilm susceptibility to antibiotics in *Pseudomonas aeruginosa*. Microorganisms. 2020;8:455. 10.3390/microorganisms8030455. 32210139 PMC7143970

[bib182] Torcato IM, Kasal MR, Brito PH et al. Identification of novel autoinducer-2 receptors in *Clostridia* reveals plasticity in the binding site of the LsrB receptor family. J Biol Chem. 2019;294:4450–63. 10.1074/jbc.RA118.006938. 30696769 PMC6433074

[bib183] Tortosa P, Logsdon L, Kraigher B et al. Specificity and genetic polymorphism of the *Bacillus* competence quorum-sensing system. J Bacteriol. 2001;183:451–60. 10.1128/JB.183.2.451-460.2001. 11133937 PMC94899

[bib184] Tripathi S, Purchase D, Govarthanan M et al. Regulatory and innovative mechanisms of bacterial quorum sensing-mediated pathogenicity: a review. Environ Monit Assess. 2022;195:75. 10.1007/s10661-022-10564-0. 36334179

[bib185] Tsumori C, Matsuo S, Murai Y et al. Quorum sensing-dependent invasion of *Ralstonia solanacearum* into *Fusarium oxysporum* chlamydospores. Microbiol Spectr. 2023;11:e0003623. 10.1128/spectrum.00036-23. 37367297 PMC10433826

[bib186] Tung TT, Jakobsen TH, Dao TT et al. Fusaric acid and analogues as Gram-negative bacterial quorum sensing inhibitors. Eur J Med Chem. 2017;126:1011–20. 10.1016/j.ejmech.2016.11.044. 28033578

[bib187] Ujita Y, Sakata M, Yoshihara A et al. Signal production and response specificity in the *phc* quorum sensing systems of *Ralstonia solanacearum* species complex. ACS Chem Biol. 2019;14:2243–51. 31513382 10.1021/acschembio.9b00553

[bib188] Valentini M, Filloux A. Multiple roles of c-di-GMP signaling in bacterial pathogenesis. Annu Rev Microbiol. 2019;73:387–406. 10.1146/annurev-micro-020518-115555. 31500536

[bib189] Vieira FJD, Nadal-Jimenez P, Teixeira L et al. *Erwinia carotovora* quorum sensing system regulates host-specific virulence factors and development delay in *Drosophila melanogaster*. mBio. 2020;11:e01292–01220. 10.1128/mBio.01292-20. 32576677 PMC7315124

[bib190] Vikram A, Jesudhasan PR, Pillai SD et al. Isolimonic acid interferes with *Escherichia coli* O157:H7 biofilm and TTSS in QseBC and QseA dependent fashion. BMC Microbiol. 2012;12:261. 10.1186/1471-2180-12-261. 23153211 PMC3562146

[bib191] Vinoj G, Vaseeharan B, Thomas S et al. Quorum-quenching activity of the AHL-lactonase from *Bacillus licheniformis* DAHB1 inhibits *Vibrio* biofilm formation in vitro and reduces shrimp intestinal colonisation and mortality. Mar Biotechnol. 2014;16:707–15. 10.1007/s10126-014-9585-9. 25060960

[bib192] Vinothkannan R, Tamizh MM, Raj CD et al. Fructose furoic acid ester: an effective quorum sensing inhibitor against uropathogenic *Escherichia coli*. Bioorg Chem. 2018;79:310–8. 10.1016/j.bioorg.2018.05.009. 29800818

[bib193] Wan X, Yang J, Ahmed W et al. Functional analysis of pde gene and its role in the pathogenesis of *Xanthomonas oryzae* pv. *oryzicola*. Infect Genet Evol. 2021;94:105008. 10.1016/j.meegid.2021.105008. 34284137

[bib194] Wang M, Li X, Song S et al. The cis-2-dodecenoic acid (BDSF) quorum sensing system in *Burkholderia cenocepacia*. Appl Environ Microbiol. 2022;88:e02342–21. 10.1128/aem.02342-21. 34985987 PMC8863054

[bib195] Wang M, Zeng J, Zhu Y et al. A 4-Hydroxybenzoic acid-mediated signaling system controls the physiology and virulence of *Shigella sonnei*. Microbiol Spectr. 2023a;11:e0483522. 10.1128/spectrum.04835-22. 37036340 PMC10269604

[bib196] Wang Q, Ji F, Guo J et al. LotS/LotR/Clp, a novel signal pathway responding to temperature, modulating protease expression via c-di-GMP mediated manner in *Stenotrophomonas maltophilia* FF11. Microbiol Res. 2018;214:60–73. 10.1016/j.micres.2018.05.014. 30031482

[bib197] Wang Y, Bian Z, Wang Y. Biofilm formation and inhibition mediated by bacterial quorum sensing. Appl Microbiol Biotechnol. 2022;106:6365–81. 10.1007/s00253-022-12150-3. 36089638

[bib198] Wang Y, Feng X, Wang W et al. Application of encapsulated quorum quenching strain *Acinetobacter pittii* HITSZ001 to a membrane bioreactor for biofouling control. Separations. 2023b;10:127. 10.3390/separations10020127.

[bib199] Wang Y, Wang F, Wang C et al. Positive regulation of spoilage potential and biofilm formation in *Shewanella baltica* OS155 via quorum sensing system composed of DKP and orphan LuxRs. Front Microbiol. 2019;10:135. 10.3389/fmicb.2019.00135. 30804914 PMC6370745

[bib200] Wen Y, Kim IH, Son JS et al. Iron and quorum sensing coordinately regulate the expression of vulnibactin biosynthesis in *Vibrio vulnificus*. J Biol Chem. 2012;287:26727–39. 10.1074/jbc.M112.374165. 22696215 PMC3411011

[bib201] Wheeler KM, Oh MW, Fusco J et al. MvfR shapes *Pseudomonas aeruginosa* interactions in polymicrobial contexts: implications for targeted quorum-sensing inhibition. Cells. 2025;14:744. 10.3390/cells14100744. 40422247 PMC12109783

[bib202] Winson MK, Swift S, Fish L et al. Construction and analysis of luxCDABE-based plasmid sensors for investigating *N*-acyl homoserine lactone-mediated quorum sensing. FEMS Microbiol Lett. 1998;163:185–92. 10.1111/j.1574-6968.1998.tb13044.x. 9673021

[bib203] Wong SY, Charlesworth JC, Benaud N et al. Communication within east antarctic soil bacteria. Appl Environ Microbiol. 2020;86:e01968–19. 10.1128/AEM.01968-19 PMC6912078 31628145

[bib204] Wu K, Zheng Y, Wu Q et al. *Vibrio parahaemolyticus* cqsA controls production of quorum sensing signal molecule 3-hydroxyundecan-4-one and regulates colony morphology. J Microbiol. 2019;57:1105–14. 10.1007/s12275-019-9379-x. 31686391

[bib205] Xie Q, Zhao A, Jeffrey PD et al. Identification of a molecular latch that regulates staphylococcal virulence. Cell Chem Biol. 2019;26:548–558.e4. 10.1016/j.chembiol.2019.01.006. 30773482 PMC6506218

[bib206] Xin C, Schauder S, Potier N et al. Structural identification of a bacterial quorum-sensing signal containing boron. Nature. 2002;415:545–9. 11823863 10.1038/415545a

[bib207] Xuan J, Feng W, Wang J et al. Antimicrobial peptides for combating drug-resistant bacterial infections. Drug Resist Updat. 2023;68:, 100954. 10.1016/j.drup.2023.100954. 36905712

[bib208] Yan J, Li P, Wang X et al. RasI/R quorum sensing system controls the virulence of *Ralstonia solanacearum* strain EP1. Appl Environ Microbiol. 2022;88:e0032522. 10.1128/aem.00325-22. 35876567 PMC9361817

[bib209] Yang Q, Zou P, Cao Z et al. QseC inhibition as a novel antivirulence strategy for the prevention of acute hepatopancreatic necrosis disease (AHPND)-causing *Vibrio parahaemolyticus*. Front Cell Infect Microbiol. 2020;10:594652. 10.3389/fcimb.2020.594652. 33553003 PMC7859628

[bib210] Yang YX, Xu ZH, Zhang YQ et al. A new quorum-sensing inhibitor attenuates virulence and decreases antibiotic resistance in *Pseudomonas aeruginosa*. J Microbiol. 2012;50:987–93. 10.1007/s12275-012-2149-7. 23274985

[bib211] Ye J, Chen X. Current promising strategies against antibiotic-resistant bacterial infections. Antibiotics. 2022;12:67. 10.3390/antibiotics12010067. 36671268 PMC9854991

[bib212] Yee DA, Niwa K, Perlatti B et al. Genome mining for unknown–unknown natural products. Nat Chem Biol. 2023;1–8. 36702957 10.1038/s41589-022-01246-6 PMC10159913

[bib213] Yeon KM, Cheong WS, Oh HS et al. Quorum sensing: a new biofouling control paradigm in a membrane bioreactor for advanced wastewater treatment. Environ Sci Technol. 2009;43:380–5. 10.1021/es8019275. 19238968

[bib214] Yi L, Dong X, Grenier D et al. Research progress of bacterial quorum sensing receptors: classification, structure, function and characteristics. Sci Total Environ. 2021;763:143031. 10.1016/j.scitotenv.2020.143031. 33129525

[bib215] Yu W, Xu L, Graham N et al. Pre-treatment for ultrafiltration: effect of pre-chlorination on membrane fouling. Sci Rep. 2014;4:6513. 10.1038/srep06513. 25269375 PMC4180805

[bib216] Yuan L, Wu H, Wang B et al. ComX improves acid tolerance by regulating the expression of late competence proteins in *Lactococcus lactis* F44. J Dairy Sci. 2021;104:9556–69. 10.3168/jds.2021-20184. 34147226

[bib217] Zaman N, Azam SS. From normal to competo-allosteric regulation: insights into the binding pattern dynamics of DSPI protein of *Pseudomonas aeruginosa*. J Biomol Struct Dyn. 2020;2:1–20. 10.1080/07391102.2020.1711805 31903856

[bib218] Zeng X, Zou Y, Zheng J et al. Quorum sensing-mediated microbial interactions: mechanisms, applications, challenges and perspectives. Microbiol Res. 2023;273:, 127414. 10.1016/j.micres.2023.127414. 37236065

[bib219] Zhang J, Yao T, Liu J et al. Recent advances in cyclodipeptide synthases-dependent biosynthetic pathway. Chin J Org Chem. 2019;39:328–38. 10.6023/cjoc201806003.

[bib220] Zhang L, Li S, Liu X et al. Sensing of autoinducer-2 by functionally distinct receptors in prokaryotes. Nat Commun. 2020;11:5371. 10.1038/s41467-020-19243-5. 33097715 PMC7584622

[bib221] Zhang Y, Qi K, Jing Y et al. LsrB-based and temperature-dependent identification of bacterial AI-2 receptor. AMB Expr. 2017;7:188. 10.1186/s13568-017-0486-y. PMC5634988 29019162

[bib222] Zhao X, Yu Z, Ding T. Quorum-sensing regulation of antimicrobial resistance in bacteria. Microorganisms. 2020;8:425. 10.3390/microorganisms8030425. 32192182 PMC7143945

[bib223] Zhou JW, Ruan LY, Chen HJ et al. Inhibition of quorum sensing and virulence in *Serratia marcescens* by hordenine. J Agric Food Chem. 2019;67:784–95. 10.1021/acs.jafc.8b05922. 30609368

[bib224] Zhou L, Zhang LH, Camara M et al. The DSF Family of quorum sensing signals: diversity, biosynthesis, and turnover. Trends Microbiol. 2017;25:293–303. 10.1016/j.tim.2016.11.013. 27979499

[bib225] Zouhir S, Perchat S, Nicaise M et al. Peptide-binding dependent conformational changes regulate the transcriptional activity of the quorum-sensor NprR. Nucleic Acids Res. 2013;41:7920–33. 10.1093/nar/gkt546. 23793817 PMC3763537

